# Targeted activation of GPER enhances the efficacy of venetoclax by boosting leukemic pyroptosis and CD8+ T cell immune function in acute myeloid leukemia

**DOI:** 10.1038/s41419-022-05357-9

**Published:** 2022-10-31

**Authors:** Jun Ren, Yonghong Tao, Meixi Peng, Qiaoling Xiao, Yipei Jing, Junpeng Huang, Jing Yang, Can Lin, Minghui Sun, Li Lei, Zesong Yang, Zailin Yang, Ling Zhang

**Affiliations:** 1 Key Laboratory of Laboratory Medical Diagnostics Designated by the Ministry of Education, School of Laboratory Medicine, Chongqing Medical University, No.1, Yixueyuan Road, Chongqing, 400016 China; 2 Department of Hematology, The First Affiliated Hospital of Chongqing Medical University, Chongqing, China; 3 Chongqing University Cancer Hospital, Chongqing, China

**Keywords:** Apoptosis, Acute myeloid leukaemia

## Abstract

Acute myeloid leukemia (AML) is a rapidly progressing and often fatal hematopoietic malignancy. Venetoclax (VEN), a recent FDA-approved BCL-2 selective inhibitor, has high initial response rates in elderly AML patients, but the majority of patients eventually acquire resistance. Multiple studies have demonstrated that the female sex is associated with better outcomes in patients with AML, which are predominantly attributed to estrogen signaling. As a novel membrane estrogen receptor, G protein-coupled estrogen receptor (GPER)-mediated-rapid estrogen effects have attracted considerable attention. However, whether targeting GPER enhances the antileukemic activity of VEN is unknown. In this study, we first demonstrated that GPER expression was dramatically reduced in AML cells owing to promoter hypermethylation. Furthermore, pharmacological activation of GPER by G-1 combined with VEN resulted in synergistic antileukemic activity in vitro and in vivo. Mechanistically, G-1/VEN combination synergistically triggered concurrent mitochondria-related apoptosis and gasdermin E (GSDME)-dependent pyroptosis by activating p38-MAPK/myeloid cell leukemia 1 (MCL-1) axis. Importantly, leukemic pyroptosis heightened CD8+ T cell immune function by releasing interleukin (IL)-1β/18 into the tumor microenvironment. Our study corroborates that GPER activation shows a synergistic antileukemic effect with VEN, making it a promising therapeutic regimen for AML.

## Introduction

Acute myeloid leukemia (AML) is a highly heterogeneous malignancy characterized by uncontrolled proliferation of myeloblasts and cessation of normal hematopoietic differentiation [1]. Chemotherapy and allogeneic stem cell transplantation remain the mainstay of treatment. However, the majority of elderly patients fail to tolerate intensive standard chemotherapy [2–4]. Recently, venetoclax (VEN), a BCL-2 selective inhibitor, was proven to be effective in inducing disease remission in older patients when used with low-dose cytarabine or hypomethylating drugs [5, 6]. Unfortunately, the majority of responses are transient and culminate in acquired resistance or refractory disease [7]. Therefore, there is an urgency to identify more effective adjuvant treatment strategies to improve the outcomes of VEN treatment in patients with AML.

The incidence and age-adjusted mortality rates for AML are lower in female patients than in male patients, suggesting that biological differences between sexes influence disease initiation, progression, and response to antileukemic therapeutics [8, 9]. It is well accepted that estrogen, a primary female hormone, is a crucial causal factor for sex-specific differences [10], and alterations in its function play key roles in the development of numerous cancers [11, 12]. This raises the question of whether estrogen affects the response to antileukemic therapy. Traditionally, estrogen functions primarily through two nuclear estrogen receptors (ERα/β) [13]. ERs are expressed at low levels due to hypermethylation in leukemia [14, 15]. ERα activation by tamoxifen activates apoptosis of MLL-AF9-induced AML cells [16] and sensitizes leukemic cells to conventional chemotherapy [17]. GPER, a recently discovered membrane estrogen receptor, mediates rapid non-genomic estrogen action [18]. Compared to ERα/β, GPER is a comprehensive and effective mechanoregulator, with more diversified action patterns. Indeed, GPER activation can trigger multiple downstream effectors, such as mitogen-activated protein kinase (MAPK) and phosphoinositide 3-kinase (PI3K) [19, 20]. Furthermore, accumulating evidence suggests that GPER-mediated estrogen signaling may be tumor suppressive, including some non-sex hormone-related cancers such as hepatocellular carcinoma [21], colon cancer [22], and pancreatic ductal adenocarcinoma [23]. In particular, activation of GPER with the selective agonist G-1 showed a synergistic effect with the BTK inhibitor ibrutinib in chemotherapy-free therapies against mantle cell lymphoma [24], and also rendered melanoma cells more vulnerable to immune checkpoint inhibitor therapy [25]. However, no data have been published thus far regarding the potential role of GPER in the antileukemic activity of VEN in AML.

For over three decades, apoptosis has been intensively investigated as a major form of regulated cell death underlying chemotherapy [26]. An increasing body of evidence suggests that VEN exerts antileukemic effects by inducing apoptosis [27], and resistance to apoptosis has been shown to play an irreplaceable role in disease recurrence [28]. Recently, many novel forms of regulated cell death, such as pyroptosis [29], ferroptosis [30], and cuproptosis [31], have been reported to be triggered in the context of multiple stimuli. Thus, from a therapeutic standpoint, novel treatment potential may exist for utilizing the nonapoptotic machinery to enhance antileukemic activity and overcome resistance to VEN therapy. Recent reports have revealed that pyroptosis induced by chemotherapeutic drugs can boost the effect of antitumor therapy [32]. Pyroptosis is a faster and more severe form of programmed cell death than apoptosis and is characterized by cell swelling and large bubbles blowing from the plasma membrane [33]. Pyroptosis was initially linked to proteolytic fragmentation of gasdermin D (GSDMD) through inflammatory caspase-1/4/5/11 [34]. Notably, recent studies have proposed the novel concept that gasdermin E (GSDME, encoded by *DFNA5*), another member of the gasdermin family, can be cleaved by active caspase-3, releasing the N-terminal effector domain, which produces pores in the cell membrane upon chemotherapy treatment [35]. Interestingly, exogenous activation of pyroptosis has been reported to elicit robust antitumor immunity [36]. The release of proinflammatory cytokines induced by pyroptosis can promote the activation of immune cells to enhance chemosensitivity [37]. It is becoming increasingly clear that the bone marrow immune environment of patients with leukemia is profoundly altered, contributing to the disease severity [38]. Therapeutic strategies aimed at improving immune cell activity in AML are therefore imperative. Encouragingly, these breakthrough reports have demonstrated that apoptosis activated by chemotherapy drugs can be switched to pyroptosis, which is closely associated with the mitochondrial pathway [39]. Given that GPER activation induces mitochondrial dysfunction [40], whether G-1 and VEN (G-1/VEN) combination treatment could trigger pyroptosis and further activate immune cells to kill leukemic cells more effectively remains to be defined.

In this study, we first determined that GPER expression was downregulated in AML, and pharmacological activation of GPER could eradicate AML cells and enhance the antileukemic activity of VEN by synergistically inducing apoptosis and pyroptosis both in vitro and in vivo. Mechanistically, G-1/VEN combination treatment promoted MCL-1 degradation via the p38-MAPK signaling, which contributed to caspase-3-dependent GSDME cleavage. Furthermore, the release of pro-inflammatory cytokines induced by GSDME-mediated pyroptosis heightened CD8+ T cell immune function in the leukemic microenvironment. Based on this information, targeted activation of GPER combined with VEN could serve as a new treatment option for patients with AML.

## Results

### GPER expression was downregulated in AML

**Fig. 1:**
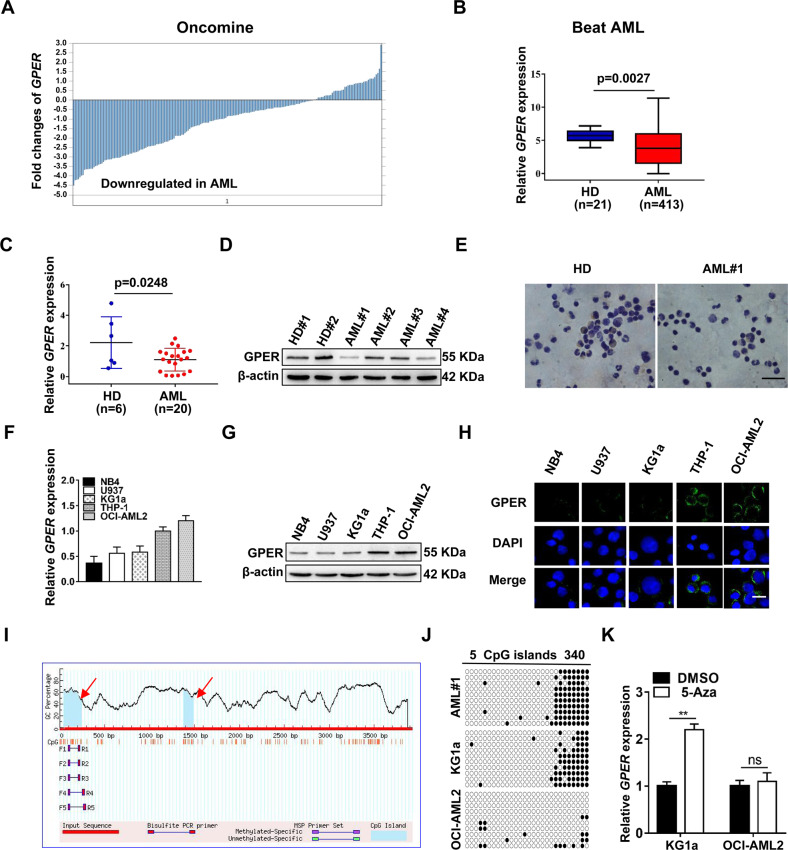
GPER expression is decreased in leukemic cells. **A**, **B** GPER levels in AML patients and HD were identified from Oncomine and Beat AML databases. **C**–**E** qRT-PCR, western blot and IHC staining analyses of GPER levels in primary AML blasts and counterparts from HD (Scale bar: 10 μm). **F**, **G** qRT-PCR and western blot analyses of GPER levels in AML cell lines. **H** The subcellular localization of GPER (green) was detected by IF staining. The nucleus was stained blue with DAPI (Scale bar: 25 μm). **I** Potential methylation sites in GPER CpG island were identified using MethPrimer software. **J** Methylation status of GPER promoter was validated by Bisulfite genomic DNA sequencing. Each black dots represented a methylated cytosine residue in the CpG islands whereas each white dots represented unmethylated CpG dinucleotides. **K** qRT-PCR analysis of GPER level in the AML cells treated with 1 μM 5-Aza for 48 h. The data are expressed as the mean ± SD (*n* = 3). **p < 0.01. ns, not significant.

To investigate GPER expression in AML, we first analyzed GPER levels using the Oncomine and Beat AML databases. The results revealed that GPER expression was considerably lower in leukemia patients than in healthy donors (HD) (Fig. 1A, B). The expression level of GPER was quantitated by qRT-PCR (Fig. 1C), western blot (Fig. 1D) and IHC staining (Fig. 1E) analyses, and GPER was found to be low expressed in newly diagnosed leukemia patients (*n* = 20) compared with HD (*n* = 6). Next, GPER expression pattern was determined in a panel of leukemia cell lines. As expected, GPER was expressed at diverse levels (Fig. 1F, G) and principally located on the cell membrane (Fig. 1H). In addition, we determined the potential mechanism underlying GPER downregulation. The methylation sites in the GPER CpG islands were identified using MethPrimer software (http://www.urogene.org/cgi-bin/methprimer/methprimer.cgi) (Fig. 1I). In support of this, the low GPER group (KG1a cells and primary AML cells) showed a higher promoter methylation level compared to the high GPER group (OCI-AML2 cells) using bisulfite genomic DNA sequencing (Fig. 1J). This was further supported by the finding that 5-Aza (a DNA methyltransferase inhibitor) treatment greatly boosted the mRNA expression of GPER in KG1a cells (Fig. 1K). These findings suggest that the expression of GPER modified by promoter methylation is downregulated in AML.

### Pharmacological activation of GPER inhibits leukemic cell survival via mitochondria-related apoptosis

**Fig. 2:**
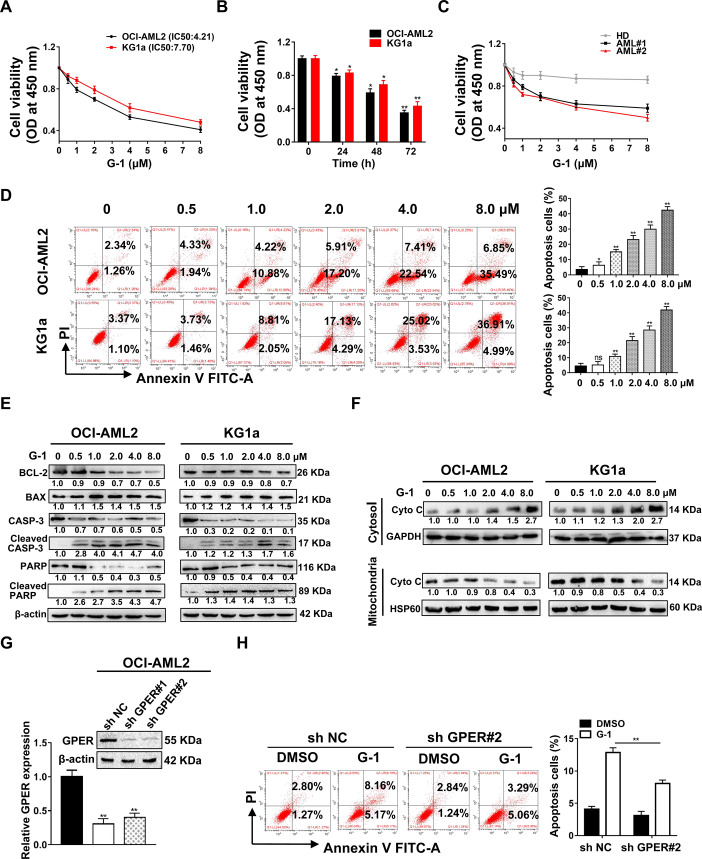
Pharmacologic activation of GPER inhibits leukemic cell survival via mitochondrial-related apoptosis. **A** CCK-8 assay of cell viability in the leukemic cells treated with corresponding concentrations of GPER agonist G-1 for 48 h, IC50 values were calculated on basis of drug concentrations that causes 50% cell viability. **B** Leukemic cells were treated with 1 μM G-1 for the corresponding times. **C** CCK-8 assay of cell viability in the primary blasts and PBMNCs from HD treated with G-1 for 48 h. **D**, **E** FCM of apoptosis in the cell lines treated with G-1 for 24 h, and western blotting of the indicated proteins. **F** Western blot analysis of Cyto C in the mitochondria fractions and cytosolic fraction of the cells treated with indicated drugs for 24 h. HSP60 or GAPDH were used as an internal control. **G** Western blot analysis of GPER in OCI-AML2 cells infected with two independent sh RNAs targeting GPER. **H** FCM of apoptosis in the cells treated with 1 μM G-1 for 24 h. The data are expressed as the mean ± SD (*n* = 3). *p < 0.05, **p < 0.01. ns, not significant.

To exploit the biological function of GPER in AML, we treated leukemic cells with the GPER agonist G-1 to determine the effects of GPER activation on cell survival. OCI-AML2 (high GPER group) and KG1a cells (low GPER group) were selected for study. The results revealed that G-1 treatment significantly inhibited the growth of leukemic cells in a concentration-(Fig. 2A) and time-(Fig. 2B) dependent manner. Notably, G-1 treatment restrained the proliferation of primary blasts, but had little effect on PBMNCs from HD (Fig. 2C). Consistent with these findings, the colony formation assay showed a lower proportion of colony forming units in the G-1 treated leukemic cells than in the DMSO group (Fig. S1A). In addition, G-1 treatment increased the percentage of G2/M cells and upregulated the levels of the cell cycle-associated protein P21, but downregulated those of CCNA2, CCND1 and C-MYC (Fig. S1B, C). G-1 treatment significantly increased the cell apoptosis rate and upregulated the levels of cell apoptosis-related proteins cleaved caspase-3(CASP-3), cleaved PARP and BAX, but downregulated those of BCL-2, CASP-3 and PARP (Fig. 2D, E). Given that apoptosis is ignited by mitochondrial membrane potential (ΔΨm), which results in the release of Cytochrome C (Cyto C), our results demonstrated that G-1 treatment decreased ΔΨm (Fig. S2A) and Cyto C expression in the mitochondrial fraction, and increased Cyto C expression in the cytosolic fraction of leukemic cells (Fig. 2F). Furthermore, G-1 treatment increased mitochondrial reactive oxygen species (ROS) (Fig. S2B). GPER knockdown (Fig. 2G) assays were performed to confirm the effect of GPER on leukemic cell survival. As anticipated, the silencing of GPER partially attenuated G-1-induced apoptosis (Fig. 2H). These data demonstrate that pharmacological activation of GPER induces mitochondria-related apoptosis in leukemic cells.

### GPER agonist G-1 and venetoclax synergistically inhibit leukemic cell survival through apoptosis and pyroptosis

**Fig. 3:**
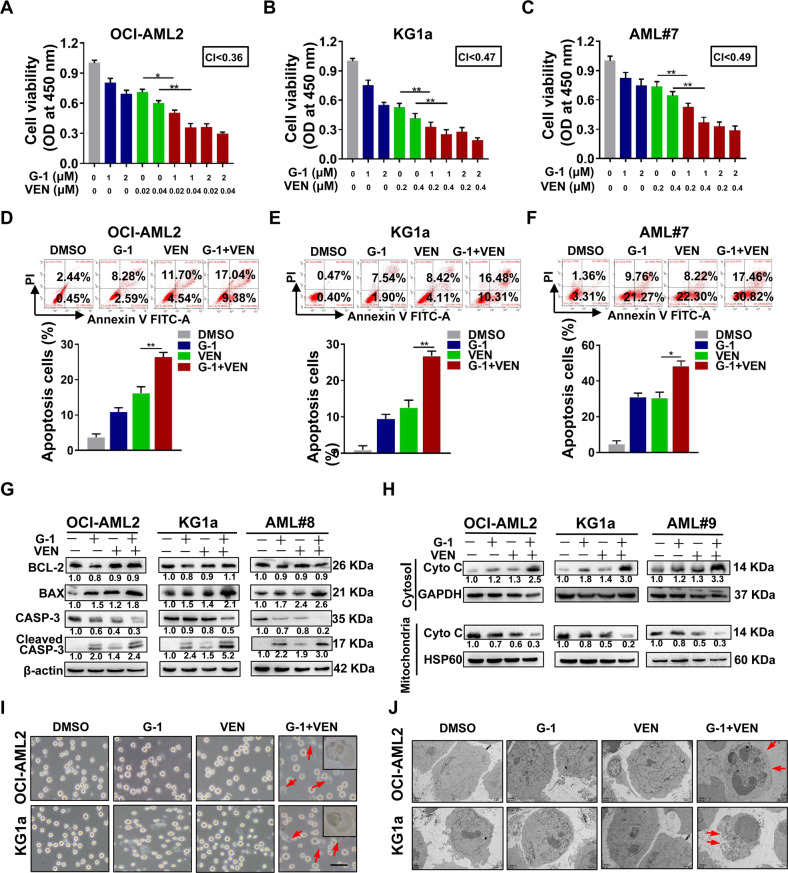
GPER agonist G-1 and venetoclax synergistically inhibit leukemic cell survival through apoptosis and pyroptosis. **A**–**C** CCK-8 assay of cell viability in the cell lines and primary blasts treated with G-1 and VEN, alone or in combination for 48 h, CI values were calculated using CompuSyn software. **D**–**F** FCM of apoptosis in the cell lines, primary blasts treated with G-1 (1 μM) and VEN (OCI-AML2; 0.02 μM, KG1a; 0.2 μM, AML#7; 0.2 μM), alone or in combination for 24 h. **G** Western blotting of these apoptosis related proteins. **H** Western blotting of Cyto C in the mitochondria fractions and cytosolic fraction of the cells treated with indicated drugs for 24 h. **I** Representative light microscopy images of the same treated cells as in Fig. 3D, E. The red arrowheads indicated the characteristic balloons on the cell membrane (Scale bar: 50 μm). **J** Representative transmission electronic micrographs of the leukemic cells (Scale bar: 2 μm). The data are expressed as the mean ± SD (*n* = 3). *p < 0.05, **p < 0.01, ***p < 0.001.

Having demonstrated GPER-mediated inhibition of cell survival, we sought to determine whether the addition of G-1 to VEN would augment its antileukemic activity. First, two leukemic cell lines and primary blasts were treated with G-1 and VEN either alone or in combination. In contrast to the effects of a single agent, G-1/VEN combination significantly reduced cell viability (combination index [CI], 0.36–0.49; Fig. 3A–C), increased cell apoptosis rate (Fig. 3D–F), upregulated the levels of cell apoptosis-related proteins BAX and cleaved CASP-3, but downregulated those of CASP-3 (Fig. 3G), while had little effect on PBMNCs from HD (Fig. S3A, B). In addition, G-1/VEN combination decreased ΔΨm (Fig. S3C–E) and Cyto C expression in the mitochondrial fraction, and increased Cyto C expression in the cytosolic fraction of leukemic cells compared to single treatments (Fig. 3H). Recent reports have shown that the intrinsic mitochondrial apoptotic pathway mediates pyroptosis, and these two processes occur simultaneously [41]. Upon G-1/VEN combination treatment, we observed that leukemic cells were swollen peripherally and had huge bubbles emanating from the cell membrane (Fig. 3I), which is a typical characteristic of pyroptosis. However, cells treated with a single drug did not show any apparent pyroptotic morphological changes. Transmission electron micrographs showed numerous pores forming on the plasma membranes of leukemic cells (Fig. 3J). Given that cytosolic components may be released after the breakdown of plasma membrane integrity, we detected pyroptosis-related indicators. The results manifested that G-1/VEN combination treatment augmented the release of LDH (Fig. S3F–H) into the leukemic cell supernatant compared to single treatments. These findings support that the GPER agonist G-1 and VEN synergistically inhibit leukemic cell survival via apoptosis and pyroptosis induction.

### GPER agonist G-1 and venetoclax synergistically improve CD8+ T cell immune function

**Fig. 4:**
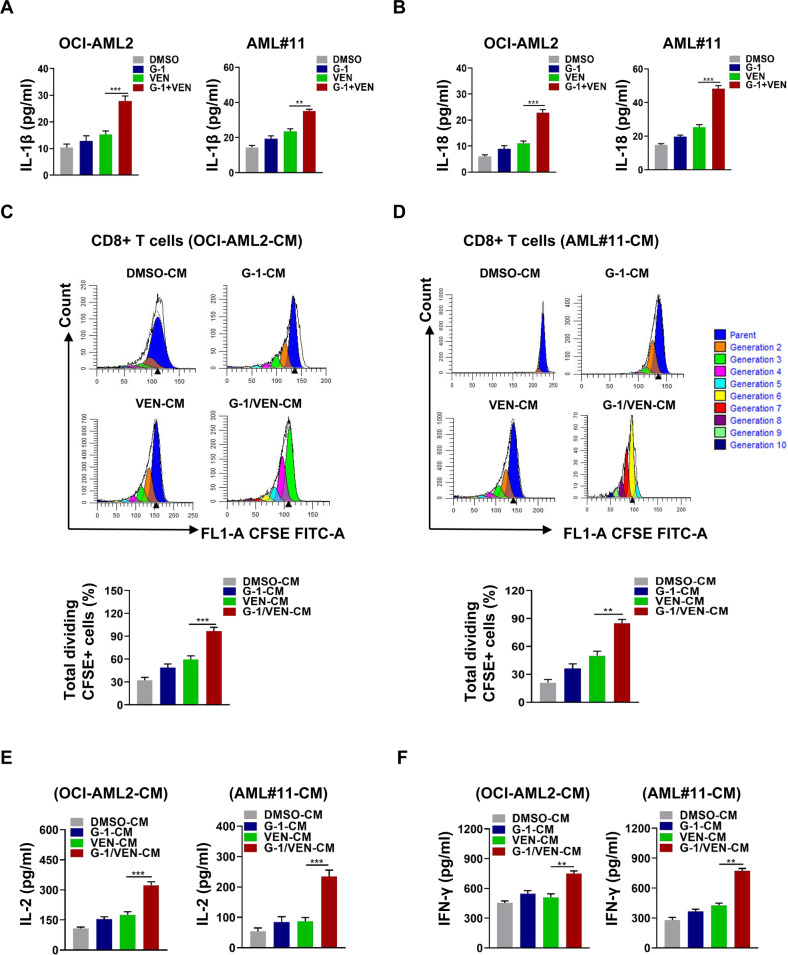
G-1 and venetoclax synergistically improve the immune function of CD8+ T cells. CD8+ T cells pre-labeled with CFSE were incubated with CM from the OCI-AML2 cells or primary blasts treated with G-1 (1 μM) and VEN (OCI-AML2; 0.02 μM, AML#11; 0.2 μM), alone or in combination for 24 h. **A**, **B** ELISA assay of IL-1β and IL-18 levels in supernatant from cell cultures. **C**, **D** FCM analysis of CD8+ T cell proliferation. **E**, **F** ELISA assay of IL-2 and IFN-γ level in supernatants from cell cultures. The data are expressed as the mean ± SD (*n* = 3). **p < 0.01, ***p < 0.001.

Given that the release of proinflammatory cytokines induced by pyroptosis can promote the activation of immune cells in the tumor microenvironment [42], we focused on examining the effect of G-1/VEN combination treatment on CD8+ T cell function in a co-culture system. The results indicated that the combination treatment increased the release of IL-1β (Fig. 4A) and IL-18 (Fig. 4B) into the leukemic cell line and primary blast supernatant compared to the single treatments. Conditioned medium (CM) from leukemic cells or primary blasts pre-treated with G-1/VEN significantly enhanced CD8+ T cell proliferation (Fig. 4C, D) and the release of IL-2 (Fig. 4E) and IFN-γ (Fig. 4F) compared to single treatment. These data suggest that G-1/VEN synergistically boosts CD8+ T cell immune function by inducing IL-1β/18 secretion via leukemic cells.

### The combination treatment induces apoptosis by downregulating MCL-1 via p38-MAPK signaling

**Fig. 5:**
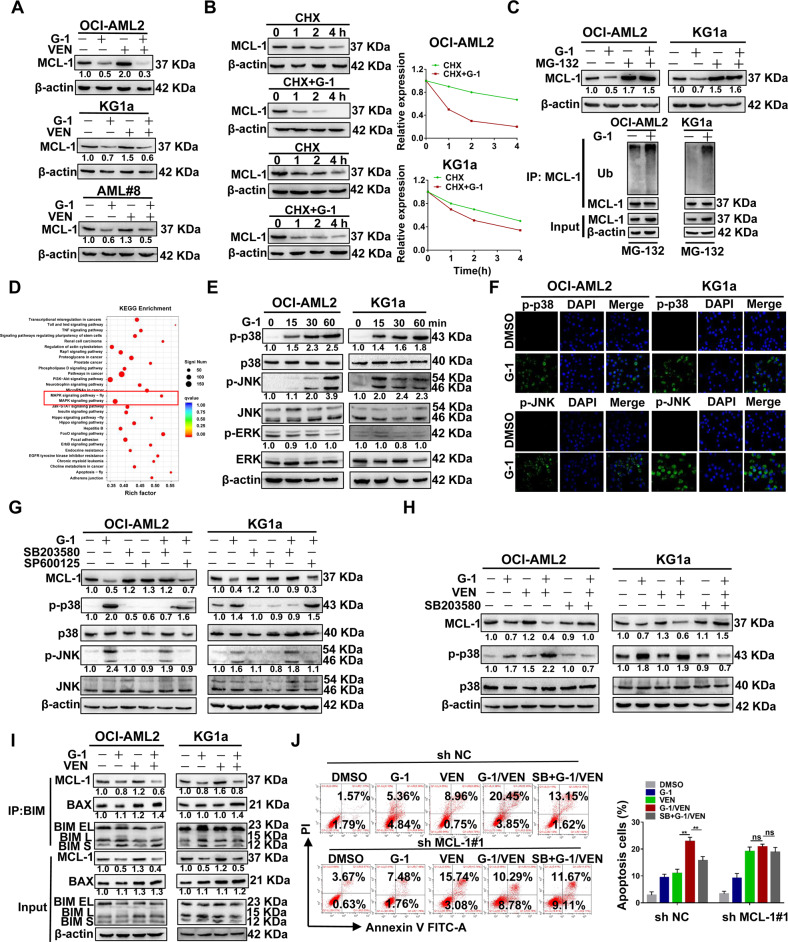
The combination treatment induces apoptosis by downregulating MCL-1 via p38-MAPK signaling. **A** Western blotting of MCL-1 in the same treated cells as in Fig. 3D–F. **B** Western blot analysis of MCL-1 in the cell lines pretreated with CHX (10 μg/ml) for 1 h and then treated with 1 μM G-1 at indicated time points. **C** Western blotting of MCL-1 in the cell lines treated with 1 μM G-1 for 24 h, with or without pretreatment with MG-132(1 μM) for 1 h. Ubiquitination analysis of the ubiquitinated MCL-1 level. **D** KEGG analysis of these significant gene signatures from corresponding NES. **E** Western blot analysis of MAPK downstream signaling in the cell lines treated with 1 μM G-1 for the corresponding times. **F** IF staining of the p-p38 and p-JNK (green). The nucleus was stained blue with DAPI (scale bar, 50 μm). **G** Western blot analysis of MCL-1 in the cell lines pretreated with 1 μM SB203580 (SB) or 5 μM SP600125 (SP) for 1 h and then exposed to 1 μM G-1 for 24 h. **H** Western blot analysis of MCL-1 in the cell lines pretreated with 1 μM SB for 1 h, followed with G-1 and VEN, alone or in combination for 24 h. **I** IP analysis followed by western blot of BIM binding proteins in the cells treated with G-1 and VEN, alone or in combination for 24 h. **J** FCM of apoptosis in the cells infected with sh RNA targeting MCL-1 pretreated with 1 μM SB for 1 h, and then exposed to G-1 and VEN, alone or in combination for 24 h. The data are expressed as the mean ± SD (*n* = 3). **p < 0.01. ns, not significant.

As MCL-1 overexpression is crucial for developing resistance to VEN in the clinic [43], we attempted to determine the effect of G-1/VEN combination treatment on MCL-1 expression in leukemic cells. As shown in Fig. 5A, combination treatment led to a significant downregulation of MCL-1 expression. However, MCL-1 mRNA levels remained unchanged upon drug treatment (Fig. S4A). Thus, we further investigated whether G-1/VEN combination is involved in regulating MCL-1 protein stability. First, protein synthesis inhibitor cycloheximide (CHX) treatment revealed that the combination reduced the half-life of MCL-1 protein (Fig. 5B). Furthermore, MCL-1 depletion induced by G-1 treatment was partially blocked by MG-132, and subsequent ubiquitination assays showed that G-1 treatment elevated the level of ubiquitinated MCL-1 (Fig. 5C). Next, we explored the specific molecular mechanisms underlying MCL-1 downregulation. RNA-sequencing was performed to compare the distinct gene profiles before and after G-1 treatment, as presented in the volcano plot (Fig. S4B). KEGG enrichment analyses demonstrated that the MAPK signaling pathway was highly enriched (Fig. 5D and Fig. S4C). It is well known that the MAPK signaling pathway includes p38, JNK and ERK cascades. In our study, the results from western blotting manifested that G-1 treatment rapidly increased the levels of phosphorylated p38 as well as JNK in a time-(Fig. 5E) and concentration-(Fig. S4D) dependent manner, but not phosphorylated ERK. This was further confirmed by immunofluorescence (Fig. 5F). Next, GPER knockdown assays were performed to confirm GPER/MAPK signaling in leukemic cells. The results indicated that the silencing of GPER attenuated the phosphorylation of p38 and JNK (Fig. S4E). MAPK inhibitors were then used to treat the leukemic cells followed by the indicated treatment. As shown in Fig. 5G, H, the p38-MAPK inhibitor SB203580 weakened the effect of G-1 or G-1/VEN combination treatment on MCL-1 expression, whereas the JNK inhibitor SP600125 did not. Based on the fact that overexpressed MCL-1 competitively binds apoptosis activator BIM to hinder its binding to apoptosis executioner BAX and prevents apoptosis [44], we further observed whether the decreased MCL-1 affects the binding of MCL-1 protein to BIM. G-1/VEN combination treatment reduced the level of MCL-1 protein and its binding to BIM in leukemic cells (Fig. 5I). Finally, rescue experiments were performed to determine whether the combination treatment facilitated leukemic cell apoptosis *via* the p38-MAPK/MCL-1 axis. MCL-1 knockdown or the p38-MAPK inhibitor SB203580 decreased apoptosis induced by the combination treatment, and the effect of SB203580 was rescued by silencing MCL-1 (Fig. 5J and Fig. S4F). These results demonstrate that the combination treatment induces apoptosis by downregulating MCL-1 via GPER/p38-MAPK signaling in leukemic cells.

### The combination treatment induces pyroptosis through triggering cleavage of GSDME via caspase-3 activation

**Fig. 6:**
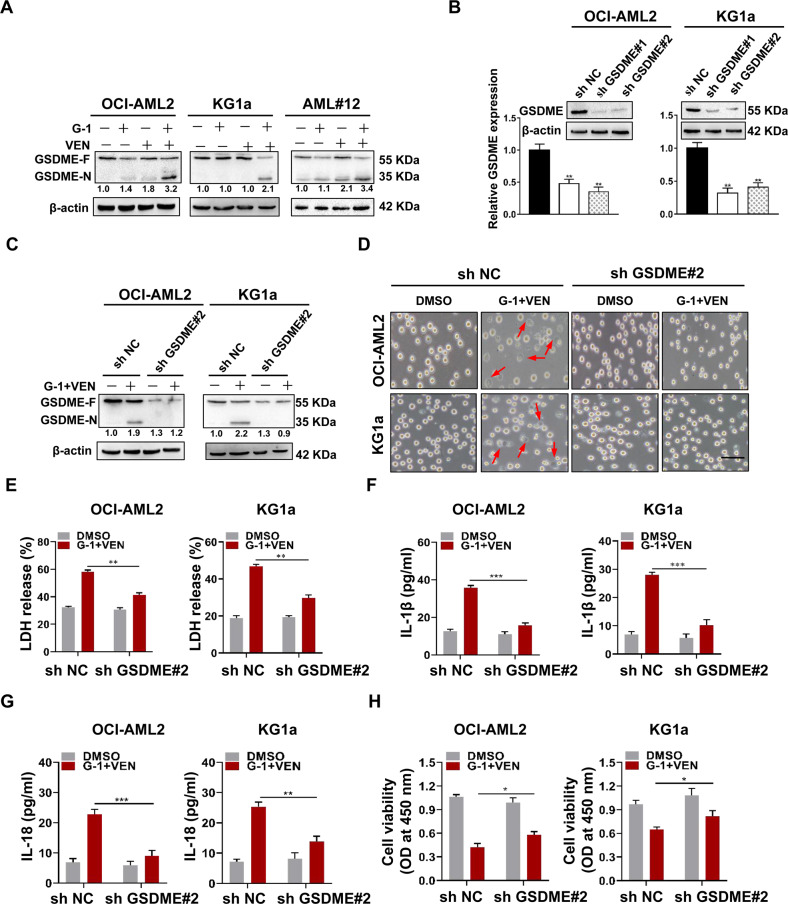
The combination treatment induces pyroptosis through triggering cleavage of GSDME. **A** Western blot analysis of GSDME-F (full-length) and GSDME-N (N-terminal fragment) in the cell lines and primary blasts treated with G-1 and VEN alone or in combination for 24 h. **B**, **C** Western blotting of GSDME and GSDME-N in the cell lines infected with two independent sh RNA targeting GSDME, and then exposed to G-1 and VEN, alone or in combination for 24 h. **D** Representative light microscopy images of the leukemic cells (Scale bar: 50 μm). **E** LDH assay of LDH level in supernatants from cell cultures. **F**, **G** ELISA assay of IL-1β and IL-18 levels in supernatants from cell cultures. **H** CCK-8 assay of cell viability in the GSDME-silenced cells treated with the indicated drugs for 48 h. The data are expressed as the mean ± SD (*n* = 3). *p < 0.05, **p < 0.01; ***p < 0.001.

In support of the mechanism of pyroptosis induced by G-1/VEN combination treatment, the expression of GSDME was detected in leukemic cells. The results of qRT-PCR (Fig. S5A) and western blotting (Fig. S5B) revealed that GSDME was expressed in the five cell lines. This was supported by the detection of GSDME expression levels in primary blasts (Fig. S5C). Notably, the G-1/VEN combination treatment increased the levels of the N-terminal fragments of GSDME (Fig. 6A) but did not influence GSDMD levels (Fig. S5D). Based on this, the effect of GSDME on the combination treatment-induced pyroptosis was clarified. GSDME knockdown (Fig. 6B) decreased the cleavage of GSDME induced by the combination treatment (Fig. 6C), and further significantly reduced plasma membrane ballooning and the secretion of LDH and IL-1β/IL-18 (Fig. 6D–G). Importantly, GSDME knockdown partially rescued cell death in response to the combination treatment (Fig. 6H). Caspase-3 is the key enzyme for GSDME cleavage [45], and the results demonstrated that G-1/VEN combination caused caspase-3 activation (Fig. S6A). Furthermore, we determined whether the combination treatment-induced pyroptosis was dependent on caspase-3 activation. The results indicated that Ac-DEVD-CHO, a caspase-3-specific inhibitor, weakened GSDME cleavage, plasma membrane ballooning, the levels of LDH and IL-1β/IL-18 release (Fig. S6B–F). Finally, we investigated whether the p38-MAPK signaling affects the combination treatment-induced pyroptosis. As expected, the p38-MAPK inhibitor SB203580 alleviated GSDME cleavage, plasma membrane ballooning, the levels of LDH and IL-1β/IL-18 release (Fig. S7A–E). Collectively, these results demonstrate that the combination treatment triggers pyroptosis by inducing the cleavage of GSDME via caspase-3 activation in leukemic cells.

### The combination treatment synergistically impairs leukemogenesis in vivo

**Fig. 7:**
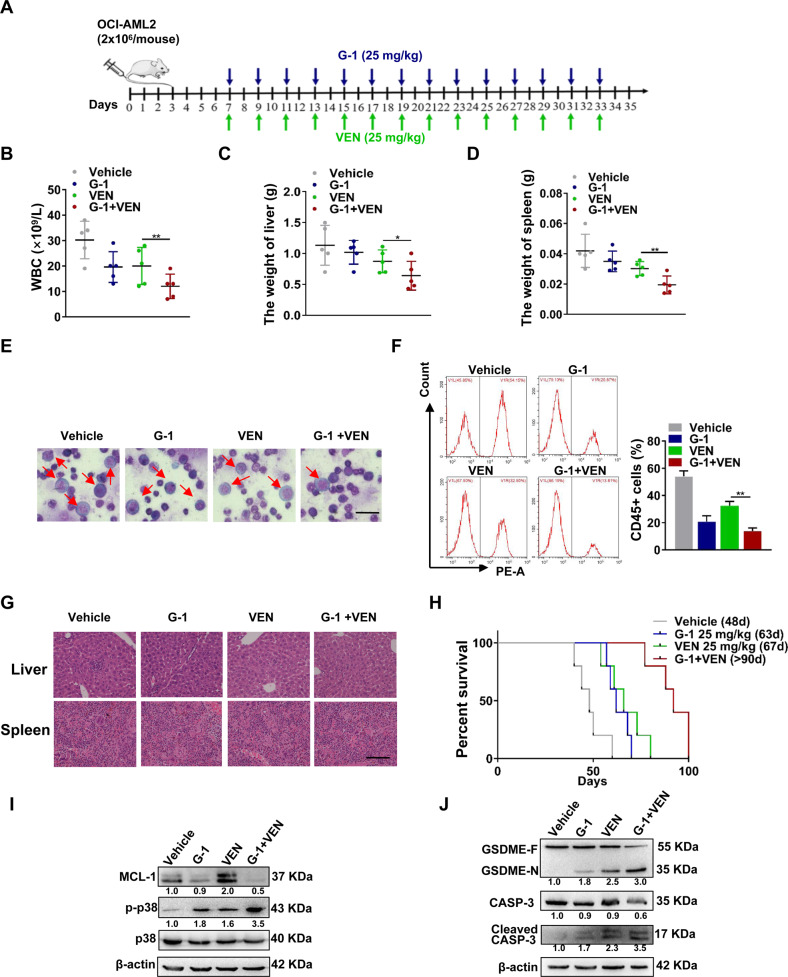
The combination treatment synergistically impairs leukemogenesis in vivo. **A** Treatment schedule of leukemia mice model. **B** The WBC count was recorded. **C**, **D** Weights of the liver and spleen of mice were measured. **E** Wright’s staining of immature cells from bone marrow (Scale bar, 25 μm). **F** FCM analysis of human CD45+ leukemic cells. **G** H&E staining of representative liver and spleen infiltration (Scale bar, 50 μm). **H** Kaplan–Meier analysis of the survival curves of the mice. **I**, **J** Western blotting of MCL-1, p-p38, GSDME-N, and cleaved CASP-3. The data are expressed as the mean ± SD (*n* = 3). *p < 0.05, **p < 0.01.

In view of the above in vitro data, we further investigated the effect of G-1/VEN combination treatment on leukemogenesis in vivo. A total of 2 × 10^6^ OCI-AML2 cells were injected into NOD/SCID mice via the tail vein. The treatment was started on day 7 and continued every other day until day 33. Fourteen injections were administered to mice receiving either G-1(25 mg/kg), VEN (25 mg/kg), or in combination (Fig. 7A). The weights of all mice were recorded before and after the injection of leukemic cells (Fig. S8A). The combination treatment resulted in lower counts of white blood cells (WBCs) (Fig. 7B) and lower weights of the liver and spleen (Fig. 7C, D and Fig. S8B). The bone marrow smear was stained using Wright’s staining and the proportion of human-CD45+ cells was measured by FCM. The results confirmed that the G-1/VEN combination treatment group had a significantly lower number of leukemic cells (Fig. 7E) and proportion of CD45+ cells (Fig. 7F) than the other groups. Additionally, H&E staining revealed less infiltration of the liver and spleen in the combination treatment group (Fig. 7G). Strikingly, Kaplan–Meier survival curves demonstrated that combination treatment significantly prolonged median survival (>90 days) compared with treatment with vehicle, G-1 or VEN (median survival, 48, 63, and 67 days, respectively; Fig. 7H). Finally, western blotting demonstrated that the combination treatment decreased the levels of MCL-1 but increased those of phosphorylated p38, cleaved caspase-3 and the N-terminal fragment of GSDME (Fig. 7I, J). These findings support that G-1/VEN combination treatment synergistically impairs leukemogenesis in vivo.

## Discussion

**Fig. 8:**
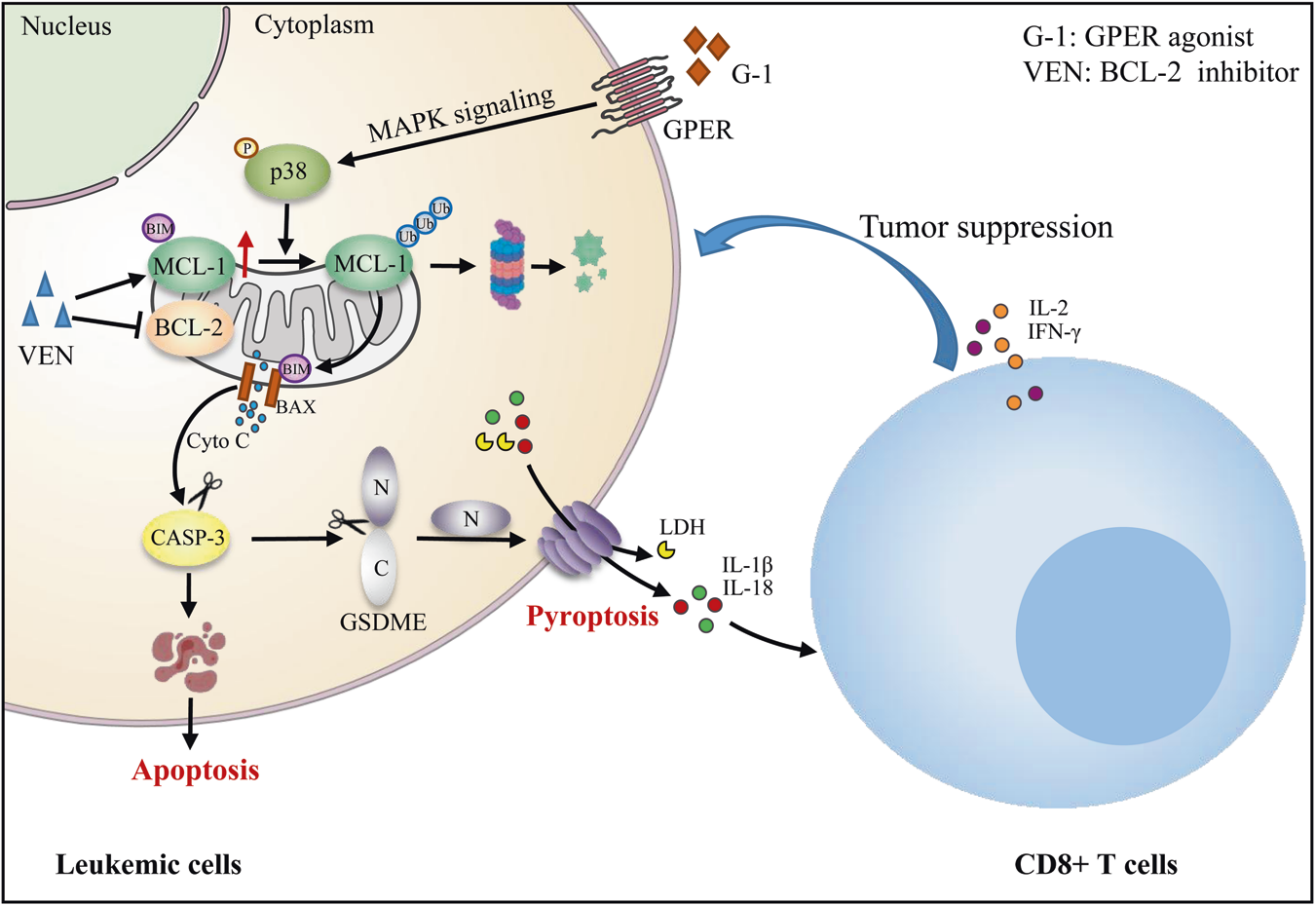
Proposed model for how targeted activation of GPER improves the antileukemic activity of venetoclax. G-1 and VEN synergistically trigger simultaneous apoptosis and GSDME-dependent pyroptosis *via* activating the p38-MAPK/MCL-1 axis in leukemic cells. Moreover, the release of IL-1β/18 induced by pyroptosis heightens the tumor suppressive function of CD8+ T cells.

Although venetoclax (VEN) is a promising agent for the treatment of acute myeloid leukemia (AML), its therapy alone fails to eliminate most leukemic cells [46]. Thus, effective combination strategies are necessary to successfully treat AML. Our data demonstrated that GPER agonist G-1 and VEN combination treatment facilitated MCL-1 degradation by activating p38-MAPK signaling, synergistically inducing leukemic apoptosis and GSDME-dependent pyroptosis both in vitro and in vivo. Moreover, pyroptosis-induced release of IL-1β/18 further boosted the immune function of CD8+ T cells in the leukemic microenvironment (Fig. 8).

In the present study, reduced GPER levels were first detected in AML cases compared to HD. Several reports have demonstrated a tumor suppressor effect of GPER in some non-sex hormone-related cancers [21–23]. Next, based on the GPER different expression and high MCL-1 levels (Data not shown), we chose OCI-AML2 and KG1a cell lines as cellular model for experiments. The results showed that GPER activation by G-1 suppressed leukemic cell survival *via* the induction of mitochondrial-related apoptosis. In fact, previous studies have demonstrated that ERβ is a therapeutic target for AML because its activation suppresses leukemic cell growth [15, 47]. Our group and others have showed that estrogen signaling has a tumor inhibitory effect on AML development. Thus, whether GPER mediated-rapid estrogen effects could promote the antileukemic activity of VEN deserves further exploration.

In this work, we explored the potential combined effect of GPER activation and VEN in AML. The results demonstrated that the GPER agonist G-1 and VEN synergistically induced mitochondrial-related apoptosis, whereas the combination approach had minimal effect on HD counterparts. In addition, we observed hallmark features of pyroptosis, including balloon-like bubbles and LDH release in G-1/VEN-treated leukemic cells. Our study first proposes that pyroptosis is involved in the combination treatment of VEN for leukemia. A recent study indicated that mitochondria-related apoptosis activated by chemotherapeutic drugs can be transformed into pyroptosis [39]. Similarly, Long et al. [48] reported that small-molecule inhibition of APE1 elicited robust cellular apoptosis, simultaneously invoking pyroptotic cell death in non-small cell lung cancer. Importantly, in our study, the synergistic effects of G-1 and VEN were further verified in mouse models. Therefore, our data demonstrated that GPER activation enhances the efficacy of VEN by synergistically activating concurrent apoptosis and pyroptosis.

Next, we focused on the molecular mechanism of apoptosis induced by the combination treatment. It has been acknowledged that MCL-1 upregulation plays a significant role in the intrinsic resistance to VEN [49]. In the current study, VEN treatment resulted in an increase of MCL-1 protein levels in leukemic cells, whereas the effect was completely abolished by G-1 treatment. Remarkably, MCL-1 knockdown abrogated the combinatorial activity, supporting that MCL-1 is an important contributor to cell apoptosis induced by G-1/VEN combination treatment. Similar to our observations, a study indicated that MCL-1 is a vital factor involved in the synergy between VEN and CDK9 inhibitor voruciclib [50]. On the other hand, we examined the potential mechanism of pyroptosis triggered by G-1/VEN combination treatment. Recent studies have reported that GSDME can switch apoptosis to pyroptosis in GSDME-high expression cells to competitively inhibit the combination of caspase-3 and apoptotic substrates [39]. The results in our work illuminated that the combination treatment induced the cleavage of GSDME, but did not influence GSDMD levels. Certainly, Yang et al. [51] have evidenced that pyridoxine selectively induces GSDME-mediated pyroptosis in AML cell lines. Conversely, Johnson et al. [52] reported that a DPP8/9 inhibitor induced GSDMD-dependent pyroptosis by activating Nlrp1b in leukemic cells. This suggests that distinct drug treatments induce pyroptosis by activating different GSDM subtypes in leukemia.

Notably, the release of proinflammatory cytokines induced by pyroptosis can promote the activation of immune cells in the tumor microenvironment [53]. However, there is limited knowledge regarding the relationship between pyroptosis and antitumor immunity in leukemia. In the current study, we first found that G-1/VEN-induced pyroptosis could augment CD8+ T cell function in a co-culture system. In line with our findings, Erkes et al. [54] demonstrated that mutant BRAF and MEK inhibitor-induced pyroptosis promoted T cell proliferation and contributed to the antitumor effects in melanoma. Notably, our previous study showed that tumor-derived extracellular vesicles inhibited CD8+ T cell immune function by suppressing creatine import, thereby enhancing the immune escape of NPM1mut leukemia [55]. Given the potent antileukemia capabilities of CD8+ T cells, contemporary immunotherapies aim to restore weakened T cell function in AML patients using a variety of strategies [56]. In fact, the activation of CD8+ T cell functions by G-1/VEN combination treatment in our study provides a new strategy for AML therapy. In addition to CD8+ T cells, pyroptosis of tumor cells may also facilitate the activation of many other immune cells in the tumor microenvironment [57], such as natural-killer and macrophage cells. Thus, further work is needed to explore the potential influence of the combination treatment on these types of immune cells in the leukemic microenvironment.

In summary, this is the first work to demonstrate the synergistic antileukemic effect between the GPER agonist G-1 and BCL-2 selective inhibitor VEN. Our findings support a potential therapeutic regimen combining estrogen signaling with chemotherapy drugs for leukemia.

## Materials and methods

### Chemicals

GPER selective agonist G-1, venetoclax, 5-Azacytidine, caspase-3 specific inhibitor Ac-DEVD-CHO and dimethyl sulfoxide (DMSO) were obtained from MCE (Monmouth Junction, NJ, USA). p38-MAPK inhibitor SB203580, JNK inhibitor SP600125 and protein synthesis inhibitor cycloheximide (CHX) were purchased from Millipore (Burlington, MA, USA). Proteasome inhibitor MG-132 was provided by Topscience (Shanghai, China).

### Gene expression analysis from databases

Gene expression levels and clinical information for AML patients were obtained from Beat AML (http://vizome.org/additional_figures_BeatAML.html, *n* = 413) and Oncomine (www.oncomine.org) databases. The expression of GPER was evaluated between AML patients and healthy donors using unpaired *t* test. Moreover, the overall survival of AML samples from databases was assessed through Kaplan–Meier method.

### Clinical sample information

The bone marrow or peripheral blood samples of 6 healthy donors and 20 newly diagnosed AML patients were provided by the First Affiliated Hospital of Chongqing Medical University. The patients’ sex and age were matched between the samples. Ethics Committee of Chongqing Medical University gave its approval to this project. No consent from the patients was needed, and data were analyzed anonymously. Patient characteristics are shown in the Table S1.

Bone marrow mononuclear cells (BMMNCs) or peripheral blood mononuclear cells (PBMNCs) were isolated by Ficoll Lymphocyte Separation Solution (Tianjin, China), and CD34+ cells (only if CD34+ content was,80%, otherwise used directly) were enriched immunomagnetically using an EasySep CD34 Human Positive Selection Kit (STEMCELL Technologies). Purity was verified by restaining isolated cells with an allophycocyanin (APC)-labeled anti-CD34 antibody (BD Biosciences) and analyzed on a BD FACSCalibur flow cytometer (BD Biosciences). Microscopy were utilized to confirm the leukemic blasts.

### Cell culture

The AML cell line OCI-AML2 was gained from Deutsche Sammlung von Mikroorganismen und Zellkulturen GmbH (DSMZ, Braunschweig, NI, Germany) and kept in MEM alpha media (Gibco, New York, USA). The NB4, U937, THP-1 and KG1a cell lines were offered by the American Type Culture Collection (ATCC, Manassas, VA, USA) and grown in RPMI-1640 media. The cell lines were supplemented with 10% fetal bovine serum (FBS) (Gibco) and 1% penicillin/streptomycin (Beyotime, Shanghai, China). Cell line characteristics are shown in the Table S2.

The CD8+ T cells were isolated from the peripheral blood mononuclear cells (PBMCs) of healthy donors. The PBMCs were extracted with HISTOPAQUE-1077 solution (Missouri, USA). The peripheral blood was gathered in anticoagulant tubes containing EDTA, and then stratified into HISTOPAQUE-1077 solutions. The PBMCs maintained at the plasma-Historopaque-1077 interface were charily shifted to a fresh tube after centrifugation at 650 × *g* for 30 min, and then washed twice with 1× PBS (Invitrogen). The CD8+ T cells were then purified using CD8 microbeads as a positive selection method (Miltenyi Biotec, USA). FCM analysis showed that the CD8+ T cells’ purity was more than 90%. The CD8+ T cells were planted and cultivated with anti-CD28 antibody (BD Bioscience, California, USA) to preserve proliferative potential for 3–5 days after anti-CD3 antibody (BD Bioscience) was appended to a 48-well plate for 24 h. The cells were grown at 37 °C in a humidity chamber including 5% CO_2_ (Thermo Fisher Scientific, Massachusetts, USA).

### Immunohistochemistry (IHC) analysis

Bone marrow mononuclear cells (BMMNCs) from healthy donors and leukemic blasts from AML patients were shifted to slides *via* cytospin, disposed with 3% H_2_O_2_ and blocked with 5% goat serum. The slides were then incubated at 4 °C overnight with primary antibodies against GPER (ab260033, 1:200, Abcam, Cambridge, UK), incubated using secondary antibody for 25 min, and stained with diaminobenzidine. Image-Pro 6.0 software was used to acquire and assess the photos (Media Cybernetics, Maryland, USA).

### Immunofluorescence (IF) staining

The leukemic cells were cytospun onto coverslips, fixed with 4% paraformaldehyde for 15 min, permeabilized with 0.3% Triton X-100 for 10 min, and then blocked for 25 min at room temperature with 5% goat serum. Primary antibodies against GPER (ab39742, 1:200, Abcam), Phospho-p38 MAPK (#4511, 1:1600, Cell Signaling Technology, USA), and Phospho-SAPK/JNK (#9255, 1:200, Cell Signaling Technology) were used to treat the cells overnight at 4 °C. The cells were then rinsed twice in PBS and treated with goat anti-rabbit secondary antibodies (Beyotime) for 30 min at room temperature, with 4′,6-diamino-2-phenylindole (DAPI, Beyotime) for nuclear counterstaining. A fluorescence microscope was used to examine the cells (Japan).

### Quantitative real‑time PCR (qRT‑PCR)

TRIzol reagent (Takara, Japan) was utilized to extract the total RNA, which was then reverse-transcribed into cDNA by the Prime-Script^TM^ RT Reagent Kit (Takara). The SYBR Green Reaction Kit was put to use to execute the qRT-PCR on a CFX Connect^TM^ real-time system (KAPA Biosystems, MA, USA). The initial denaturation was performed for 30 s at 95 °C, amplificated by 39 cycles of 5 s at 95 °C, 30 s at 58 °C, 20 s at 72 °C, and lastly 10 min at 72 °C for the extension. The 2^-ΔΔCt^ technique was used to calculate the relative expression of the listed genes using β-actin as an endogenous control. Each reaction was duplicated three times. The primers used for each gene are presented in Table S3.

### Bisulfite genomic DNA sequencing

The PureLink Genomic DNA Mini Kit (Invitrogen) was used to isolate genomic DNA from leukemic cells, which were then treated with sodium bisulfite using EZ DNA Methylation-Gold^TM^ (ZYMO, RESEARCH, D5005) following the instructions (Sangon Biotech, Shanghai, China). PCR primers were utilized to amplify the products and the bisulfite-modified regions of the GPER promoter were recognized. The primer sequences are listed: forward 5′-TTGGAGGTGTTTGAGGATTGAGGAA-3′, reverse 5′-ACATTCAAACCAAAAACCCTCA -3′.

### Cell infection and transfection

The lentivirus-based short hairpin RNA (sh RNA) had the following target sequences: sh GPER#1: 5′-AGTACGTGATCGGCCTGTT-3′; sh GPER#2: 5′-CGCTCCCTGCAAGCAGTCTTT-3′; sh GSDME#1: 5′-CTGGAGACTGGTAGCTTATTA-3′; sh GSDME#2: 5′-AGCTGGGAGATTAATCAACCA-3′; sh MCL-1#1: 5′-AAAAGCTTCCCTTGTACAGTA-3′; sh MCL-1#2: 5′-GATTATCTCTCGGTACCTT TT-3′. The sh RNA vectors were provided by Genechem (Shanghai, China). Leukemic cells (1 × 10^5^/well) were seeded on 24-well plates and then infected with the aforementioned lentivirus for 48 h in the context of HitransG P (Genechem), after which, they were selected for 7 d with 2 μg/mL puromycin (Sigma-Aldrich). Puromycin-resistant cells were extracted and cultured in order to conduct additional research.

### Western blotting

Whole-cell extracts were prepared in RIPA buffer (Beyotime) containing protease inhibitors. Mitochondrial and Cytosolic protein were separated using a Mitochondria Isolation Kit (Beyotime) according to the manufacture’s instructions. Next, the protein concentration was determined by BSA assay (Beyotime), and 50 μg of protein was separated on a sodium dodecyl sulfate-polyacrylamide gel electrophoresis (SDS-PAGE) and deposited onto polyvinylidene fluoride (PVDF) membranes (Bio-Rad). The membrane was sealed for 1 h at room temperature in the 5% non-fat milk (Boster Biological Technology, Wuhan, China) and incubated with primary antibodies overnight at 4 °C. The antibodies used in this study were as follows: GPER (ab260033, 1:1000), Cyclin A2 (ab181591, 1:2000), Cyclin D1 (ab226977, 1:500), GSDMD (ab209845, 1:1000), GSDME (ab215191, 1:1000), GAPDH (ab8245, 1:1000), HSP60 (ab190828, 1:1000) were all from Abcam (UK); P21 (#2947, 1:1000), C-MYC (#5605, 1:1000), BAX (#5023, 1:1000), BCL-2 (#4223, 1:1000), PARP (#9532, 1:1000), Caspase-3 (#9662, 1:1000), Cytochrome C (#11940, 1:1000), p38 MAPK (#8690, 1:1000), Phospho-p38 MAPK (#4511, 1:1000), SAPK/JNK (#9252, 1:1000), Phospho-SAPK/JNK (#9255, 1:2000), p44/42 MAPK (Erk1/2) (#4695, 1:1000), Phospho-p44/42MAPK (Erk1/2) (#4370, 1:2000), MCL-1 (#94296, 1:1000), BIM (#2933,1:1000), ubiquitin (#3936, 1:1000) were acquired from Cell Signaling Technology; β-actin (#TA-09, 1:1000) was obtained from ZSGB-BIO (Beijing, China). Lastly, PVDF membranes were incubated with the appropriate HRP-conjugated anti-rabbit or anti-mouse IgG (ZSGBBIO) for 1 h. β-actin was selected as a loading control.

### Ubiquitination assays

Leukemic cells were treated with 1 μM MG-132 for 24 h before harvesting. Next, the cells were rinsed in PBS and lysed on ice for 25 min with IP lysis buffer (Beyotime). The lysates were centrifuged at 13,300 rpm for 30 min at 4 °C. To normalize the total levels of the inputs, the BSA assay was selected to determine total cell lysates. Thereafter, the supernatant was treated with the Protein A/G Beads (Bimake) and covered with anti-MCL-1 antibody (#94296, 1:200, Cell Signaling Technology) at 4 °C overnight. The Beads were rinsed twice with PBST and boiled in 2 × SDS-PAGE (Beyotime). Proteins were then incubated with anti-ubiquitin antibody (#3936,1:1000, Cell Signaling Technology).

### Immunoprecipitation (IP) analysis

Harvested cells were cleaned twice with PBS, sonicated in IP buffer (Beyotime), then centrifuged at 13,300 rpm at 4 °C for 30 min. The levels of identified proteins were examined using the indicated antibodies to normalize the input. Briefly, clarified lysates were incubated with primary antibodies or an isotype-matched negative control IgG. Thereafter, the sample-antibody mixtures were rotated together with Protein A/G Beads (Bimake) overnight at 4 °C, cleaned three times with IP Lysis Buffer and gathered by magnetic separation. After boiling in 2×SDS-PAGE (Beyotime), proteins were subjected to western blot analysis.

### Cell proliferation assays

The CCK-8 Kit (Solarbio, Beijing, China) was used to assess cell viability. In a 96-well plate, cells (2 × 10^4^–2 × 10^5^/well) were seeded and treated with appropriate drug dosages for 24–72 h. The plate was incubated at 37 °C for an appropriate time in the dark after being added with 10 μL CCK-8 solution. The OD value at 450 nm was determined using a microplate reader (BioTeck, CA, USA). To test the cell colony formation ability, 1 × 10^3^/well cells were seeded in a 24-well plate following G-1 or DMSO treatment and cultivated in RPMI 1640 medium with 10% FBS. The colony forming-units were counted 7 d later by an inverted microscope.

### Flow cytometry (FCM) analysis

For cell cycle assessment, 1 × 10^6^ cells were fixed in 75% ethanol at 4 °C overnight. After centrifugation, the cell supernatant was removed, then RNase A was added in the dark and incubated at 37 °C for 25 min, and finally added with Propidium (PI, Millipore). The cells were cultured in the dark at 4 °C for 25 min, and the cell cycle was evaluated by FACSCalibur^TM^ Flow cytometry (BD Biosciences).

The Annexin-V and DAPI double staining was utilized to measure cell apoptosis. In a nutshell, leukemic cells were collected and washed with Annexin-V binding buffer (BD Biosciences), followed by being resuspended in the same buffer containing Annexin-V APC (BD Biosciences). Afterwards, the cells were cultured in the dark at room temperature for 10 min, finally resuspended in a buffer. DAPI (Sigma-Aldrich) was applied prior to assessment by FACSCalibur™ Flow cytometry.

Cells cultivated in 6-well plates were treated with the prescribed drug for 24 h, then incubated with an equal amount of JC-1 staining solution (Beyotime) at 37 °C for 20 min, after which the ΔΨm was assessed by FCM. Cells were stained with 5 μM DCFH-DA (Beyotime) for 25 min at 37 °C in the dark and examined with a CytoFLEX flow cytometer to quantify ROS (Beckman, California, USA).

The CellTrace^TM^ CFSE Cell Proliferation Kit (Life Technologies, California, USA) was used to determine the viability of the CD8+ T cells. The cells were first resuspended in CFSE (5 mM) buffer, cultivated at room temperature for 10 min, and then washed twice with medium. The labeled cells were planted at 1 × 10^6^ cells per well in a 48-well round-bottom plate and subsequently activated with anti-human CD28/CD3 antibodies. Cells were evaluated using a FACSCalibur™ instrument (Becton Dickinson, New Jersey, USA).

### Transmission electron microscopy

To determine the morphology of the pyroptotic cells, leukemic cells (1 × 10^6^/well) were seeded in six-well plates upon drug treatment for 24 h, fixed with ice-cold 4% glutaraldehyde for 24 h at 4 °C, and detected by a transmission electron microscope (Hitachi 7500, Tokyo, Japan).

### Lactate dehydrogenase (LDH) release assay

After the relevant drug treatments, cell culture supernatants were harvested and LDH activity was measured by the LDH Assay Kit (Beyotime). A total of 120 μL supernatant per well was transferred to a new 96-well plate, and each well was filled with 60 μL LDH detection reagent. The plates were then incubated in the dark for 30 min at room temperature. A microplate reader (BioTeck, CA, USA) was used to determine OD value at 490 nm. All samples were tested in triplicate.

### Enzyme-linked immunosorbent assay (ELISA) analysis

The levels of IL-1β and IL-18 after the indicated drug treatment were measured using the Human IL-1β ELISA Kit (NEOBIOSCIENCE, Shenzhen, China) and Human IL-18 ELISA Kit (NEOBIOSCIENCE) according to the recommended protocol. Moreover, CD8+ T cell culture supernatants were collected and the levels of IL-2 and IFN-γ were quantified by Human IL-2 ELISA Kit (NEOBIOSCIENCE) and Human IFN-γ ELISA Kit (NEOBIOSCIENCE) following the manufacturers’ instructions. Each sample was tested in triplicate.

### Animal experiments

The Animal Resources Center (Canning Vale WA, Australia) provided /five-week-old female NOD/SCID mice. Mice were injected with OCI-AML2 cells (2 × 10^6^ cells/mouse) through the tail vein and randomly assigned to one of four groups (*n* = 5): vehicle, 25 mg/kg G-1, 25 mg/kg VEN, or 25 mg/kg G-1/25 mg/kg VEN on day 7. The drugs were dissolved in 10% DMSO, 5% Tween-80, 40% PEG300 and sterile water, and were administered intraperitoneally once every 2 days. Every other day, the weights and status of the mice were assessed. FCM was used to examine human CD45+ cells. Wright’s staining was used to examine immature human leukemic cells from mouse bone marrow. The livers and spleens were removed and sectioned into 4 μm-thick sections, which were then stained with hematoxylin and eosin (H&E). The survival curves of mice were analyzed by the Kaplan–Meier method. The bone marrow cells were harvested from femurs of mice for western blot analysis. Animal experiments were approved by the Animal Care Ethics Committee of Chongqing Medical University.

### Statistical analysis

Data from three independent experiments are shown as the mean ± standard deviation (SD). One-way analysis of variance was utilized to assess the differences between three or more groups. Unpaired Student’s *t*-test was used to compare the differences between the two groups. To compare survival differences, both the Kaplan-Meier estimator and log-rank test were utilized. Statistical analyses were performed using GraphPad Prism 8.0. (*p < 0.05, **p < 0.01, ***p < 0.001, and ns indicates no significant difference).

## Supplementary information

Supplementary materials

aj-checklist

Original Data File

## Data Availability

Scripts and additional data related to this work will be available upon request to the lead contact.

## References

[1] Döhner H, Estey EH, Amadori S, Appelbaum FR, Büchner T, Burnett AK (2010). Diagnosis and management of acute myeloid leukemia in adults: recommendations from an international expert panel, on behalf of the European LeukemiaNet. Blood..

[2] Burnett A, Wetzler M, Löwenberg B (2011). Therapeutic advances in acute myeloid leukemia. J Clin Oncol.

[3] Newell LF, Cook RJ (2021). Advances in acute myeloid leukemia. BMJ..

[4] Eisfeld AK, Kohlschmidt J, Mrózek K, Blachly JS, Walker CJ, Nicolet D (2018). Mutation patterns identify adult patients with de novo acute myeloid leukemia aged 60 years or older who respond favorably to standard chemotherapy: an analysis of Alliance studies. Leukemia..

[5] DiNardo CD, Pratz K, Pullarkat V, Jonas BA, Arellano M, Becker PS (2019). Venetoclax combined with decitabine or azacitidine in treatment-naive, elderly patients with acute myeloid leukemia. Blood..

[6] DiNardo CD, Jonas BA, Pullarkat V, Thirman MJ, Garcia JS, Wei AH (2020). Azacitidine and venetoclax in previously untreated acute myeloid leukemia. N. Engl J Med.

[7] Short NJ, Konopleva M, Kadia TM, Borthakur G, Ravandi F, DiNardo CD (2020). Advances in the treatment of acute myeloid leukemia: new drugs and new challenges. Cancer Discov.

[8] Yi M, Li A, Zhou L, Chu Q, Song Y, Wu K (2020). The global burden and attributable risk factor analysis of acute myeloid leukemia in 195 countries and territories from 1990 to 2017: estimates based on the global burden of disease study 2017. J Hematol Oncol.

[9] Wiernik PH, Sun Z, Cripe LD, Rowe JM, Fernandez HF, Luger SM (2021). Prognostic effect of gender on outcome of treatment for adults with acute myeloid leukaemia. Br J Haematol.

[10] Teoh JP, Li X, Simoncini T, Zhu D, Fu X (2020). Estrogen-mediated gaseous signaling molecules in cardiovascular disease. Trends Endocrinol Metab.

[11] Liang J, Shang Y (2013). Estrogen and cancer. Annu Rev Physiol.

[12] Roberts RD (2019). Is estrogen the answer for osteosarcoma?. Cancer Res.

[13] Nilsson S, Koehler KF, Gustafsson JÅ (2011). Development of subtype-selective oestrogen receptor-based therapeutics. Nat Rev Drug Discov.

[14] Agrawal S, Unterberg M, Koschmieder S, zur Stadt U, Brunnberg U, Verbeek W (2007). DNA methylation of tumor suppressor genes in clinical remission predicts the relapse risk in acute myeloid leukemia. Cancer Res.

[15] Roma A, Spagnuolo PA (2020). Estrogen receptors alpha and beta in acute myeloid leukemia. Cancers.

[16] Sánchez-Aguilera A, Arranz L, Martín-Pérez D, García-García A, Stavropoulou V, Kubovcakova L (2014). Estrogen signaling selectively induces apoptosis of hematopoietic progenitors and myeloid neoplasms without harming steady-state hematopoiesis. Cell Stem Cell.

[17] Adachi K, Honma Y, Miyake T, Kawakami K, Takahashi T, Suzumiya J (2016). Tamoxifen enhances the differentiation-inducing and growth-inhibitory effects of all-trans retinoic acid in acute promyelocytic leukemia cells. Int J Oncol.

[18] Prossnitz ER, Arterburn JB (2015). International Union of Basic and Clinical Pharmacology. XCVII. G Protein-Coupled Estrogen Receptor and Its Pharmacologic Modulators. Pharm Rev.

[19] Prossnitz ER, Barton M (2011). The G-protein-coupled estrogen receptor GPER in health and disease. Nat Rev Endocrinol.

[20] Feldman RD, Limbird LE (2017). GPER (GPR30): a nongenomic receptor (GPCR) for steroid hormones with implications for cardiovascular disease and cancer. Annu Rev Pharm Toxicol.

[21] Qiu YA, Xiong J, Fu Q, Dong Y, Liu M, Peng M (2021). GPER-induced ERK signaling decreases cell viability of hepatocellular carcinoma. Front Oncol.

[22] Liu Q, Chen Z, Jiang G, Zhou Y, Yang X, Huang H (2017). Epigenetic down regulation of G protein-coupled estrogen receptor (GPER) functions as a tumor suppressor in colorectal cancer. Mol Cancer.

[23] Natale CA, Li J, Pitarresi JR, Norgard RJ, Dentchev T, Capell BC (2020). Pharmacologic activation of the G protein-coupled estrogen receptor inhibits pancreatic ductal adenocarcinoma. Cell Mol Gastroenterol Hepatol.

[24] Zhou L, Yu T, Yang F, Han J, Zuo B, Huang L (2021). G protein-coupled estrogen receptor agonist G-1 inhibits mantle cell lymphoma growth in preclinical models. Front Oncol.

[25] Natale CA, Li J, Zhang J, Dahal A, Dentchev T, Stanger BZ (2018). Activation of G protein-coupled estrogen receptor signaling inhibits melanoma and improves response to immune checkpoint blockade. Elife..

[26] Carneiro BA, El-Deiry WS (2020). Targeting apoptosis in cancer therapy. Nat Rev Clin Oncol.

[27] Thijssen R, Diepstraten ST, Moujalled D, Chew E, Flensburg C, Shi MX (2021). Intact TP-53 function is essential for sustaining durable responses to BH3-mimetic drugs in leukemias. Blood..

[28] Nechiporuk T, Kurtz SE, Nikolova O, Liu T, Jones CL, D’Alessandro A (2019). The TP53 apoptotic network is a primary mediator of resistance to BCL2 inhibition in AML cells. Cancer Discov.

[29] Vande Walle L, Lamkanfi M (2016). Pyroptosis. Curr Biol.

[30] Jiang X, Stockwell BR, Conrad M (2021). Ferroptosis: mechanisms, biology and role in disease. Nat Rev Mol Cell Biol.

[31] Tang D, Chen X, Kroemer G (2022). Cuproptosis: a copper-triggered modality of mitochondrial cell death. Cell Res.

[32] Zhang X, Zhang P, An L, Sun N, Peng L, Tang W (2020). Miltirone induces cell death in hepatocellular carcinoma cell through GSDME-dependent pyroptosis. Acta Pharm Sin B..

[33] Shi J, Gao W, Shao F (2017). Pyroptosis: Gasdermin-Mediated Programmed Necrotic Cell Death. Trends Biochem Sci.

[34] Shi J, Zhao Y, Wang K, Shi X, Wang Y, Huang H (2015). Cleavage of GSDMD by inflammatory caspases determines pyroptotic cell death. Nature..

[35] Wang Y, Gao W, Shi X, Ding J, Liu W, He H (2017). Chemotherapy drugs induce pyroptosis through caspase-3 cleavage of a gasdermin. Nature..

[36] Zhang Z, Zhang Y, Xia S, Kong Q, Li S, Liu X (2020). Gasdermin E suppresses tumour growth by activating anti-tumour immunity. Nature..

[37] Peng Z, Wang P, Song W, Yao Q, Li Y, Liu L (2020). GSDME enhances cisplatin sensitivity to regress non-small cell lung carcinoma by mediating pyroptosis to trigger antitumor immunocyte infiltration. Signal Transduct Target Ther.

[38] Vago L, Gojo I (2020). Immune escape and immunotherapy of acute myeloid leukemia. J Clin Invest.

[39] Rogers C, Fernandes-Alnemri T, Mayes L, Alnemri D, Cingolani G, Alnemri ES (2017). Cleavage of DFNA5 by caspase-3 during apoptosis mediates progression to secondary necrotic/pyroptotic cell death. Nat Commun.

[40] Wei W, Chen ZJ, Zhang KS, Yang XL, Wu YM, Chen XH (2014). The activation of G protein-coupled receptor 30 (GPR30) inhibits proliferation of estrogen receptor-negative breast cancer cells in vitro and in vivo. Cell Death Dis.

[41] Lu H, Zhang S, Wu J, Chen M, Cai MC, Fu Y (2018). Molecular targeted therapies elicit concurrent apoptotic and GSDME-dependent pyroptotic tumor cell death. Clin Cancer Res.

[42] Hage C, Hoves S, Strauss L, Bissinger S, Prinz Y, Pöschinger T (2019). Sorafenib induces pyroptosis in macrophages and triggers natural killer cell-mediated cytotoxicity against hepatocellular carcinoma. Hepatology..

[43] Zhang Q, Riley-Gillis B, Han L, Jia Y, Lodi A, Zhang H (2022). Activation of RAS/MAPK pathway confers MCL-1 mediated acquired resistance to BCL-2 inhibitor venetoclax in acute myeloid leukemia. Signal Transduct Target Ther.

[44] Li X, Su Y, Hege K, Madlambayan G, Edwards H, Knight T (2021). The HDAC and PI3K dual inhibitor CUDC-907 synergistically enhances the antileukemic activity of venetoclax in preclinical models of acute myeloid leukemia. Haematologica..

[45] Zhou B, Zhang JY, Liu XS, Chen HZ, Ai YL, Cheng K (2018). Tom20 senses iron-activated ROS signaling to promote melanoma cell pyroptosis. Cell Res.

[46] Konopleva M, Pollyea DA, Potluri J, Chyla B, Hogdal L, Busman T (2016). Efficacy and biological correlates of response in a phase II study of venetoclax monotherapy in patients with acute myelogenous leukemia. Cancer Discov.

[47] Rota SG, Roma A, Dude I, Ma C, Stevens R, MacEachern J (2017). Estrogen receptor β is a novel target in acute myeloid leukemia. Mol Cancer Ther.

[48] Long K, Gu L, Li L, Zhang Z, Li E, Zhang Y (2021). Small-molecule inhibition of APE1 induces apoptosis, pyroptosis, and necroptosis in non-small cell lung cancer. Cell Death Dis.

[49] Niu X, Zhao J, Ma J, Xie C, Edwards H, Wang G (2016). Binding of released Bim to Mcl-1 is a mechanism of intrinsic resistance to ABT-199 which can be overcome by combination with daunorubicin or cytarabine in AML cells. Clin Cancer Res.

[50] Luedtke DA, Su Y, Ma J, Li X, Buck SA, Edwards H (2020). Inhibition of CDK9 by voruciclib synergistically enhances cell death induced by the Bcl-2 selective inhibitor venetoclax in preclinical models of acute myeloid leukemia. Signal Transduct Target Ther.

[51] Yang W, Liu S, Li Y, Wang Y, Deng Y, Sun W (2020). Pyridoxine induces monocyte-macrophages death as specific treatment of acute myeloid leukemia. Cancer Lett.

[52] Johnson DC, Taabazuing CY, Okondo MC, Chui AJ, Rao SD, Brown FC (2018). DPP8/DPP9 inhibitor-induced pyroptosis for treatment of acute myeloid leukemia. Nat Med.

[53] Du T, Gao J, Li P, Wang Y, Qi Q, Liu X (2021). Pyroptosis, metabolism, and tumor immune microenvironment. Clin Transl Med.

[54] Erkes DA, Cai W, Sanchez IM, Purwin TJ, Rogers C, Field CO (2020). Mutant BRAF and MEK inhibitors regulate the tumor immune microenvironment via pyroptosis. Cancer Discov.

[55] Peng M, Ren J, Jing Y, Jiang X, Xiao Q, Huang J (2021). Tumour-derived small extracellular vesicles suppress CD8+ T cell immune function by inhibiting SLC6A8-mediated creatine import in NPM1-mutated acute myeloid leukaemia. J Extracell Vesicles.

[56] Daver N, Alotaibi AS, Bücklein V, Subklewe M (2021). T-cell-based immunotherapy of acute myeloid leukemia: current concepts and future developments. Leukemia.

[57] Fu C (2020). Gasdermin: a novel therapeutic target for tumour treatment by activating anti-tumour immunity. Signal Transduct Target Ther.

