# Supplementary material for: Targeted activation of GPER enhances the efficacy of venetoclax by boosting leukemic pyroptosis and CD8+ T cell immune function in acute myeloid leukemia
Source: Cell Death Dis. 2022 Oct 31;13(10):915. doi: 10.1038/s41419-022-05357-9 (PMC9622865; doi:10.1038/s41419-022-05357-9)
Supplement: Supplementary file 1 — Supplementary materials [file 41419_2022_5357_MOESM1_ESM.docx]

**Table S1.** **Clinical characteristics of primary AML patient samples.**

| **Patient** | **Gender** | **Age (year)** | **Disease status** | **FAB subtype** | **Blast purity (%)** | **Gene mutation** |
| --- | --- | --- | --- | --- | --- | --- |
| AML#1 | Female | 39 | Newly diagnosed | M2 | 27.97 | WT1 |
| AML#2 | Female | 82 | Newly diagnosed | M5 | 91.31 | NPM1,WT1 |
| AML#3 | Male | 34 | Newly diagnosed | M5 | 79.5 | MLL-AF9, FLT3-ITD |
| AML#4 | Male | 38 | Newly diagnosed | M4 | 7.78 | WT1 |
| AML#5 | Male | 40 | Newly diagnosed | M1 | 69 | NA |
| AML#6 | Female | 52 | Newly diagnosed | M4 | 16.67 | WT1, NRAS |
| AML#7 | Female | 35 | Newly diagnosed | M4 | 49 | WT1, CEBPA |
| AML#8 | Male | 89 | Newly diagnosed | M5 | 82 | NA |
| AML#9 | Male | 67 | Newly diagnosed | M5 | 9.6 | NPM1,DNMT3A |
| AML#10 | Female | 67 | Newly diagnosed | M1 | 92 | NPM1, FLT3-ITD, WT1 |
| AML#11 | Female | 56 | Newly diagnosed | M5 | 91.1 | NPM1, FLT3-ITD, WT1 |
| AML#12 | Male | 51 | Newly diagnosed | M2 | 88.5 | NA |
| AML#13 | Female | 30 | Newly diagnosed | M3 | 94.0 | PML-RARα |
| AML#14 | Female | 30 | Newly diagnosed | M5 | 60 | NPM1, FLT3-ITD |
| AML#15 | Female | 74 | Newly diagnosed | M1 | 79.5 | NA |
| AML#16 | Female | 48 | Newly diagnosed | M5 | 45 | DNMT3A |
| AML#17 | Male | 55 | Newly diagnosed | M5 | 29.75 | WT1 |
| AML#18 | Male | 69 | Newly diagnosed | M2 | 88 | NA |
| AML#19 | Male | 50 | Newly diagnosed | M3 | 61.31 | PML-RARα |
| AML#20 | Male | 57 | Newly diagnosed | M1 | 66.0 | NA |

NA: not available

**Table S2. Characteristics of the AML cell lines used in this study.**

| **Cell line** | **Gender** | **Age (year)** | **Disease status** | **FAB subtype** | **Gene fusion/mutation** |
| --- | --- | --- | --- | --- | --- |
| OCI-AML2 | Male | 65 | At diagnosis | M4 | DNMT3A R635W |
| KG1a | Male | 59 | At relapse |  | FGFR1OP2-FGFR1 |
| NB4 | Female | 23 | At relapse | M3 | PML-RARA |
| THP-1 | Male | 1 | At diagnosis | M5 | KMT2A-MLLT3 |
| U937 | Male | 37 | Refractory | M5 | CALM-AF10 |

**Table S3. Primers used for qRT-PCR.**

Abbreviations: F stands for forward; R stands for reverse

| Genes | Sequences (5' - 3') |
| --- | --- |
| *GPER* | F: 5'-CGTCATTCCAGACAGCACCGAG -3' |
| *MCL-1* | R: 5'-CGAGGAGCCAGAAGCCACATC -3'  F: 5'-AGTTAAACAAAGAGGCTGGGATGGGT-3' |
|  | R: 5'-GCCAAACCAGCTCCTACTCCAGC -3' |
| *DFNA5* | F: 5'-TGCCTACGGTGTCATTGAGTT -3' |
|  | R: 5'-TCTGGCATGTCTATGAATGCAAA -3' |
| *β-actin* | F: 5'-TGACGTGGACATCCGCAAAG -3' |
|  | R: 5'-CTGGAAGGTGGACAGCGAGG -3' |


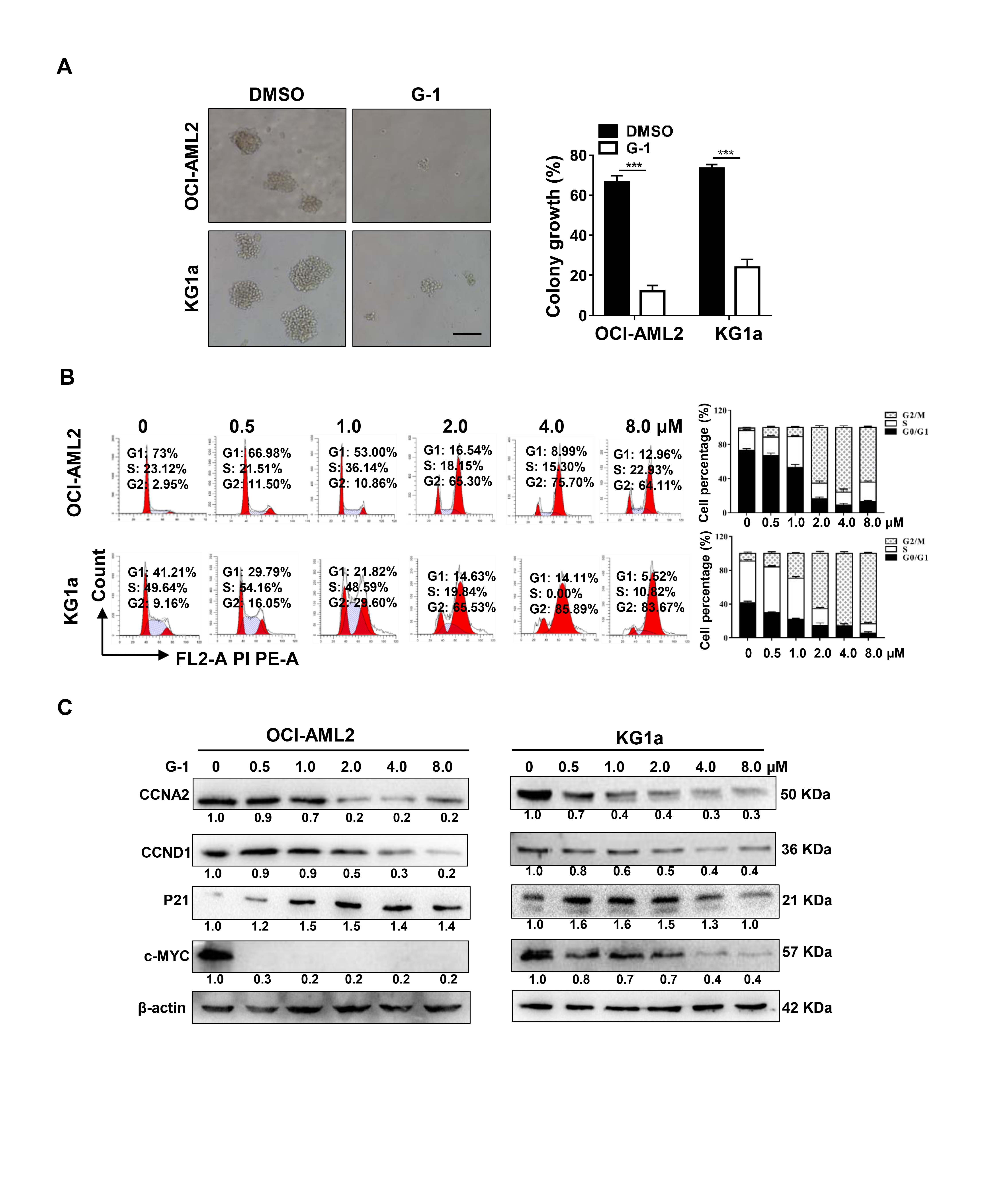


**Fig S1.** **Activation of GPER decreases the number of colony forming units and increases the percentage of G2/M cells.** (A) The number of colony forming units was counted in the cells treated with 1 μM G-1 (Scale bar: 50 μm). (B, C) FCM of cell cycle in the cells treated G-1 for 24 h, and western blot analysis of the corresponding proteins. The data are expressed as the mean ± SD (n = 3). *** *p* < 0.001.


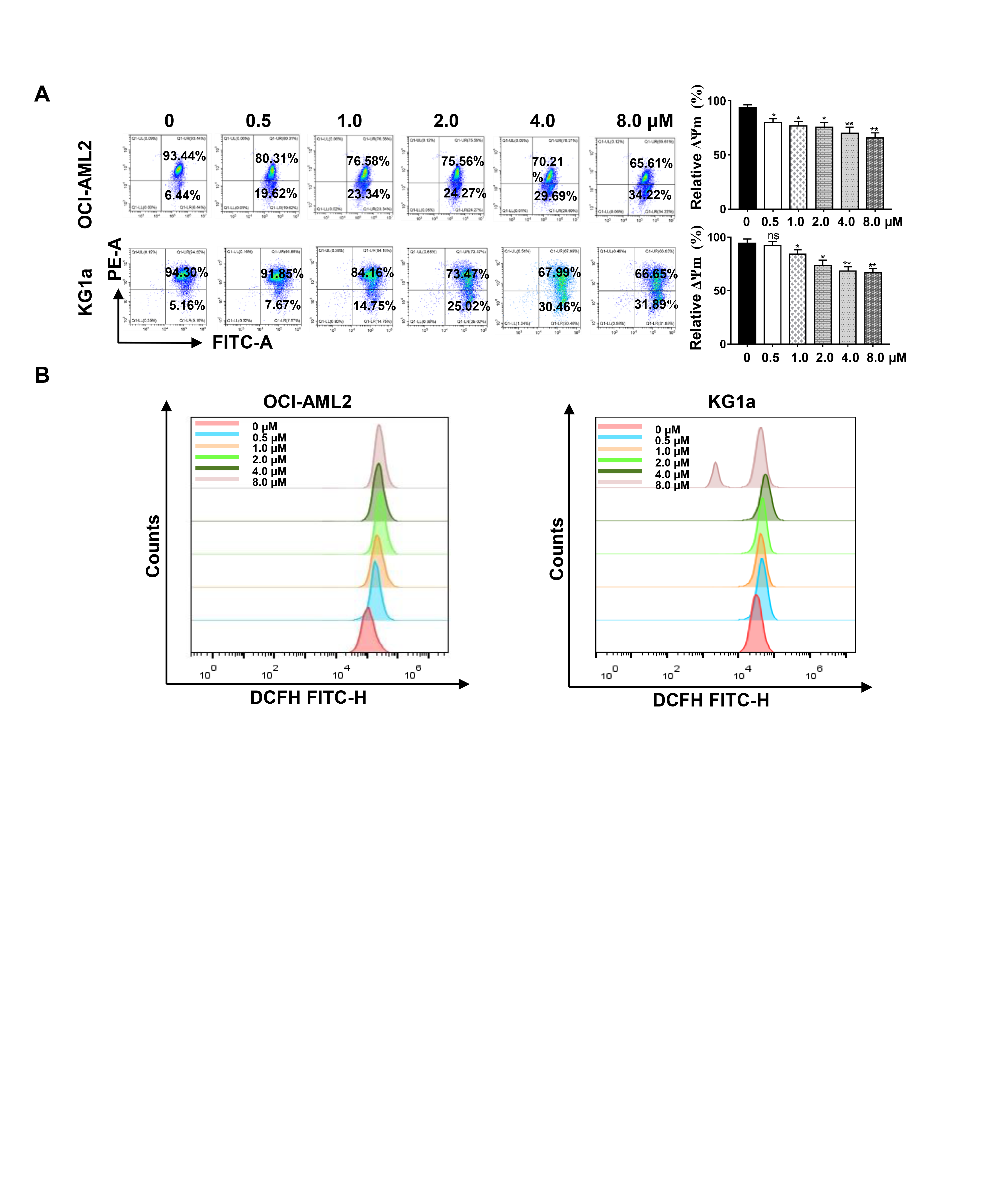


**Fig S2. Activation of GPER deceases ΔΨm and increases mitochondrial ROS generation in leukemic cells.** (A, B) FCM of ΔΨm and ROS in the cell lines treated with G-1 for 24 h. The data are expressed as the mean ± SD (n = 3). * *p* < 0.05, ** *p* < 0.01. ns, not significant.


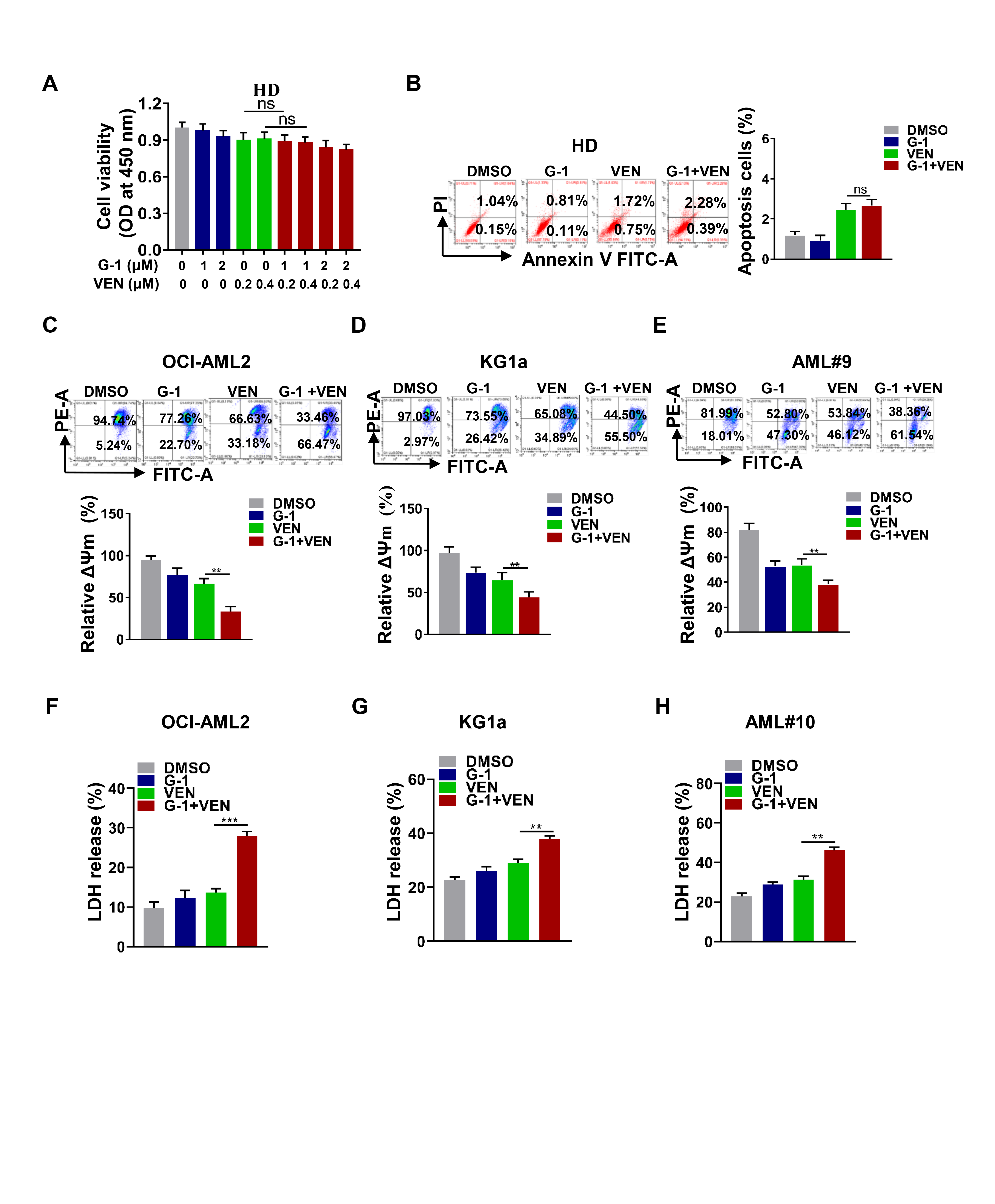


**Fig S3. GPER agonist G-1 and venetoclax synergistically inhibit leukemic cell survival through apoptosis and pyroptosis.** (A) CCK-8 assay of cell viability in the normal PBMNCs treated with G-1 and VEN, alone or in combination for 48 h. (B) FCM of apoptosis in the normal PBMNCs treated with G-1 and VEN, alone or in combination for 24 h. (C-E) FCM of ΔΨm in the cell lines and primary blasts treated with G-1 and VEN, alone or in combination for 24 h. (F-H) LDH assay of LDH levels in supernatants from cell cultures of each group. The data are expressed as the mean ± SD (n = 3). * *p* < 0.05, ** *p* < 0.01. ns, not significant.


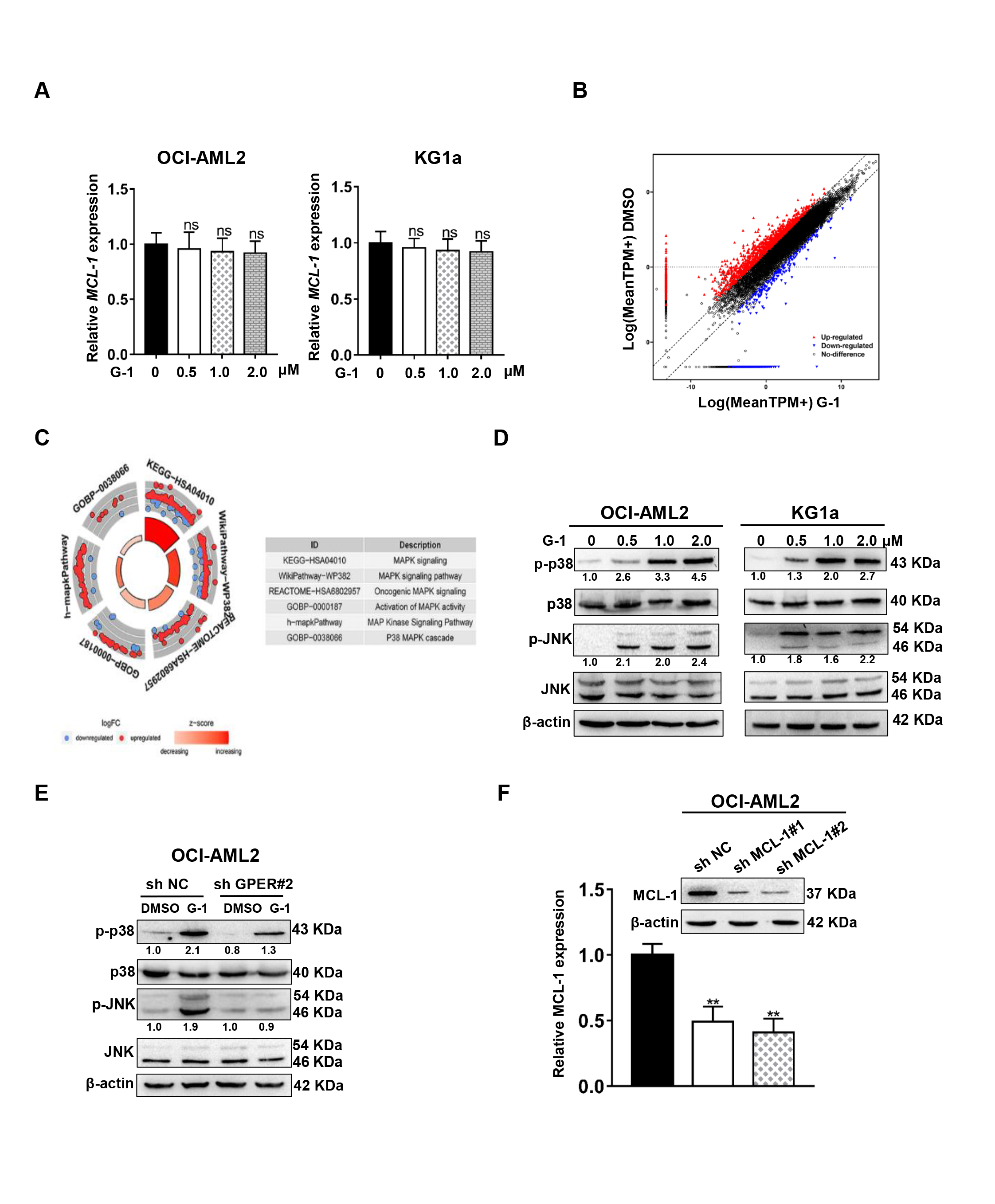


**Fig S4. The combination treatment induces apoptosis by downregulating MCL-1 *via* p38-MAPK signaling.** (A) qRT-PCR analysis of MCL-1 level in the cell lines treated with G-1 for the corresponding concentrations. (B) Volcano plot was used to visualize the differentially expressed genes of leukemic cells exposed to G-1 vs DMSO for 24 h. The red/green dots represented the upregulated or downregulated genes. (C) Significantly aberrant canonical pathways were identified using the DAVID database. (D) Western blotting of p-p38 and p-JNK in the cell lines treated with G-1 for the corresponding times and concentrations. (E) Western blotting of p-p38 and p-JNK in the leukemic cells infected with sh RNA targeting GPER followed with 1 μM G-1 for 24 h. (F) Western blotting of MCL-1 level in leukemic cells infected with sh RNA targeting MCL-1. The data are expressed as the mean ± SD (n = 3). ns, not significant.


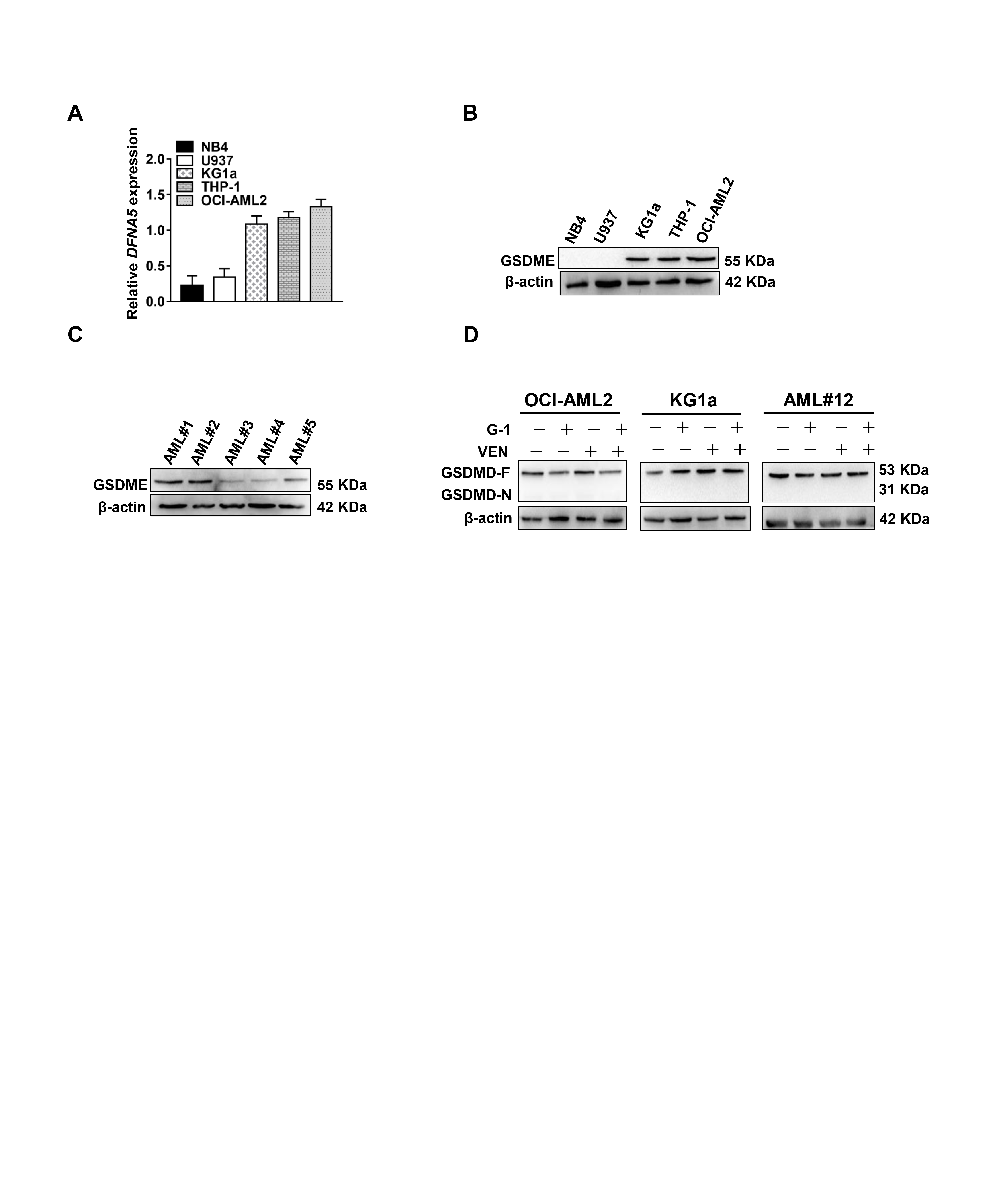


**Fig S5. GSDME, but not GSDMD, is cleaved to induce pyroptosis in leukemic cells.** (A-C) qRT-PCR and western blot analyses of GSDME level in cell lines and primary blasts. (D) Western blot analysis of GSDMD-F and GSDMD-N in the cell lines and primary blasts treated with G-1 and VEN, alone or in combination for 24 h. The data are expressed as the mean ± SD (n = 3).


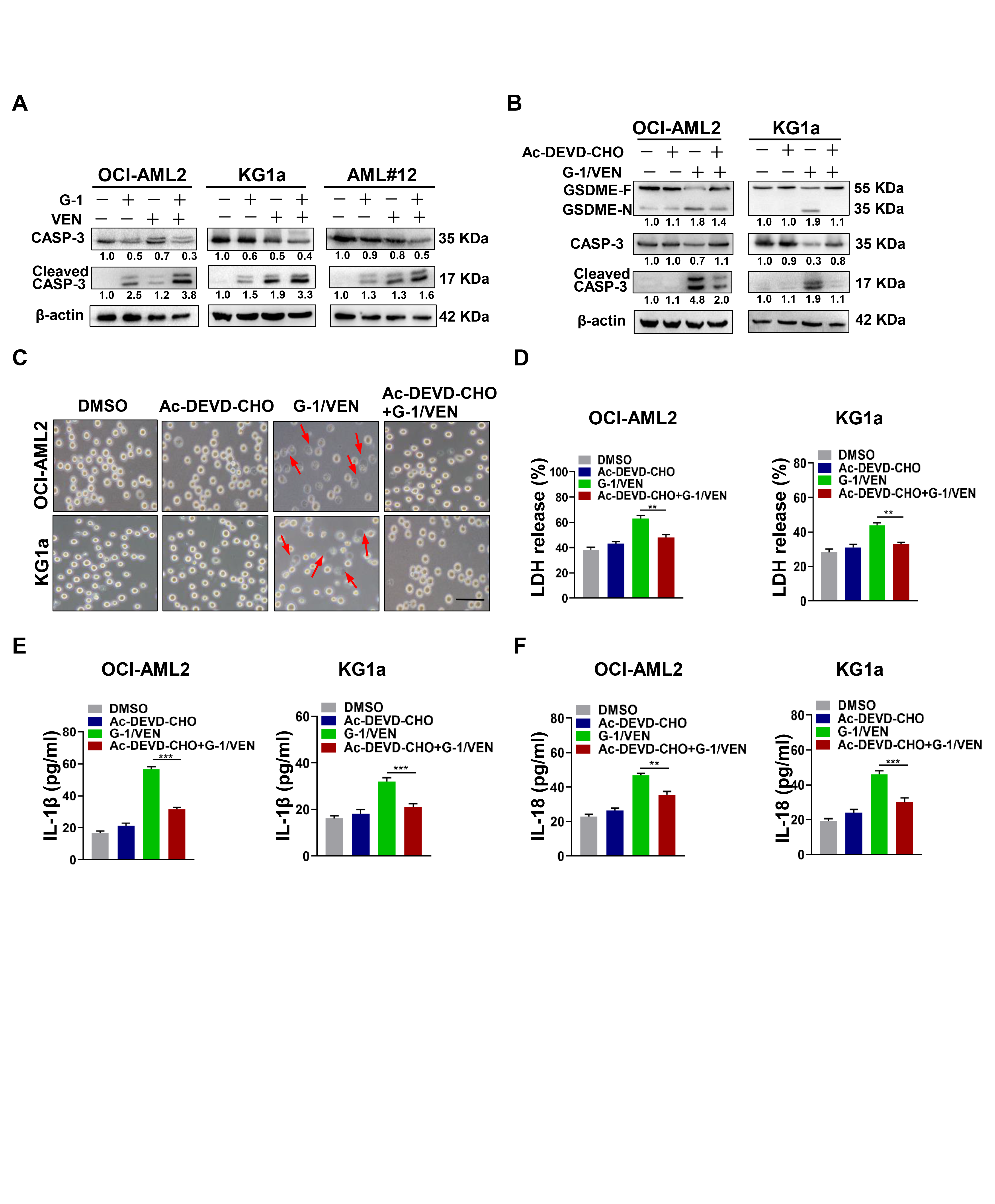


**Fig S6.** **Caspase-3 is required for the cleavage of GSDME in G-1/VEN-induced pyroptosis in leukemic cells.** (A) Western blotting of caspase-3 (CASP-3) and cleaved CASP-3 in the cell lines treated with G-1 and VEN, alone or in combination for 24 h. (B) Western blotting of GSDME-N and cleaved CASP-3 levels in the cells pretreated with 10 μM Ac-DEVD-CHO for 1 h, and then exposed to G-1 and VEN in combination for 24 h. (C) Representative light microscopy images of the leukemic cells (Scale bar: 50 μm). (D) LDH assay of LDH level in supernatants from cell cultures of each group. (E, F) ELISA assay of IL-1β and IL-18 levels in supernatants from cell cultures of each group. The data are expressed as the mean ± SD (n = 3). ** *p* < 0.01; *** *p* < 0.001.


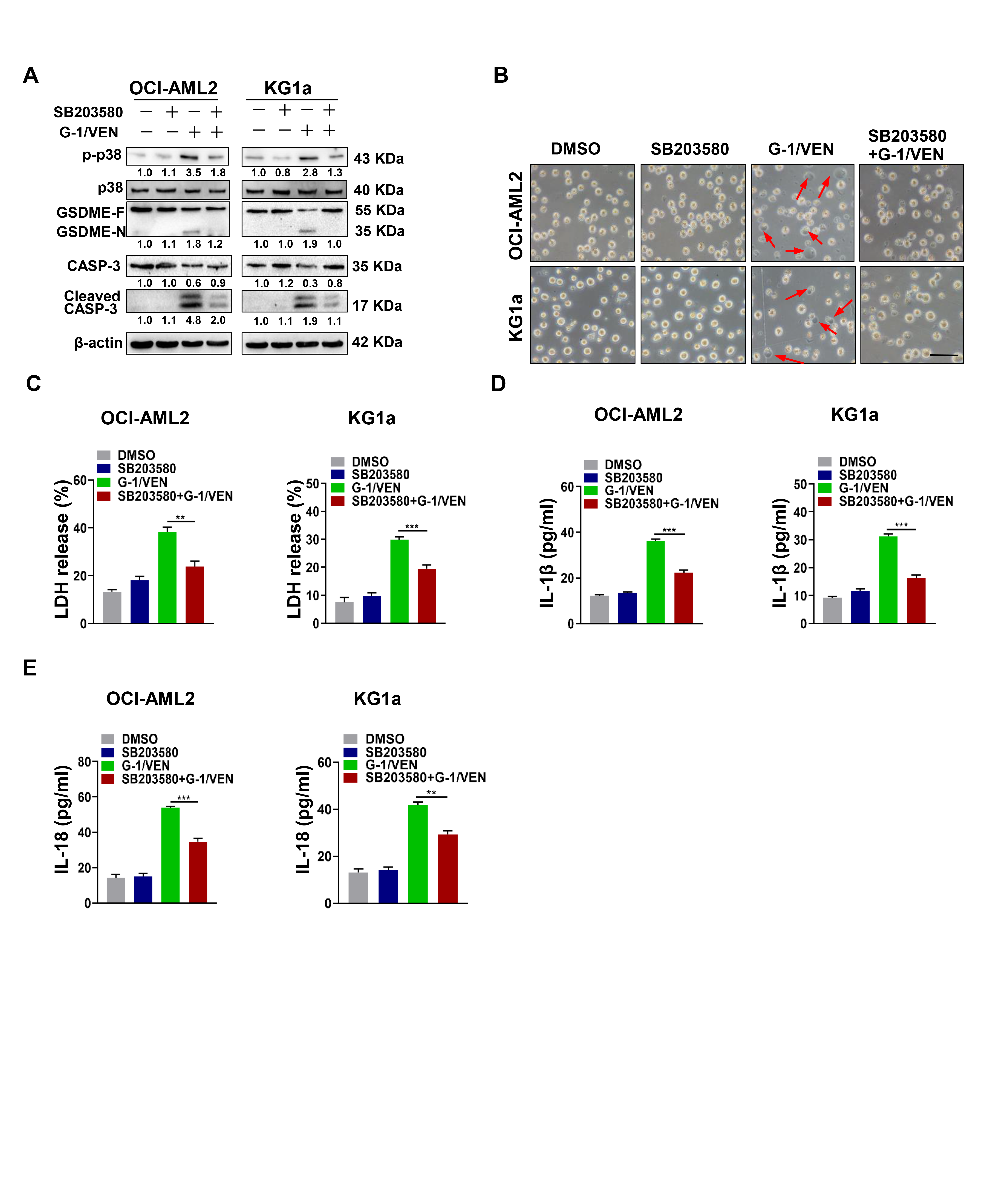


**Fig S7.** **The combination treatment induces GSDME-dependent pyroptosis *via* p38-MAPK/caspase-3 activation**. (A) Western blotting of GSDME-N and cleaved CASP-3 levels in the cells pretreated with 10 μM SB203580 for 1 h, and then exposed to G-1 and VEN in combination for 24 h. (B) Representative light microscopy images of the leukemic cells (Scale bar: 50 μm). (C) LDH assay of LDH level in supernatants from cell cultures of each group. (D, E) ELISA assay of IL-1β and IL-18 levels in supernatants from cell cultures of each group. The data are expressed as the mean ± SD (n = 3). ** *p* < 0.01; *** *p* < 0.001.


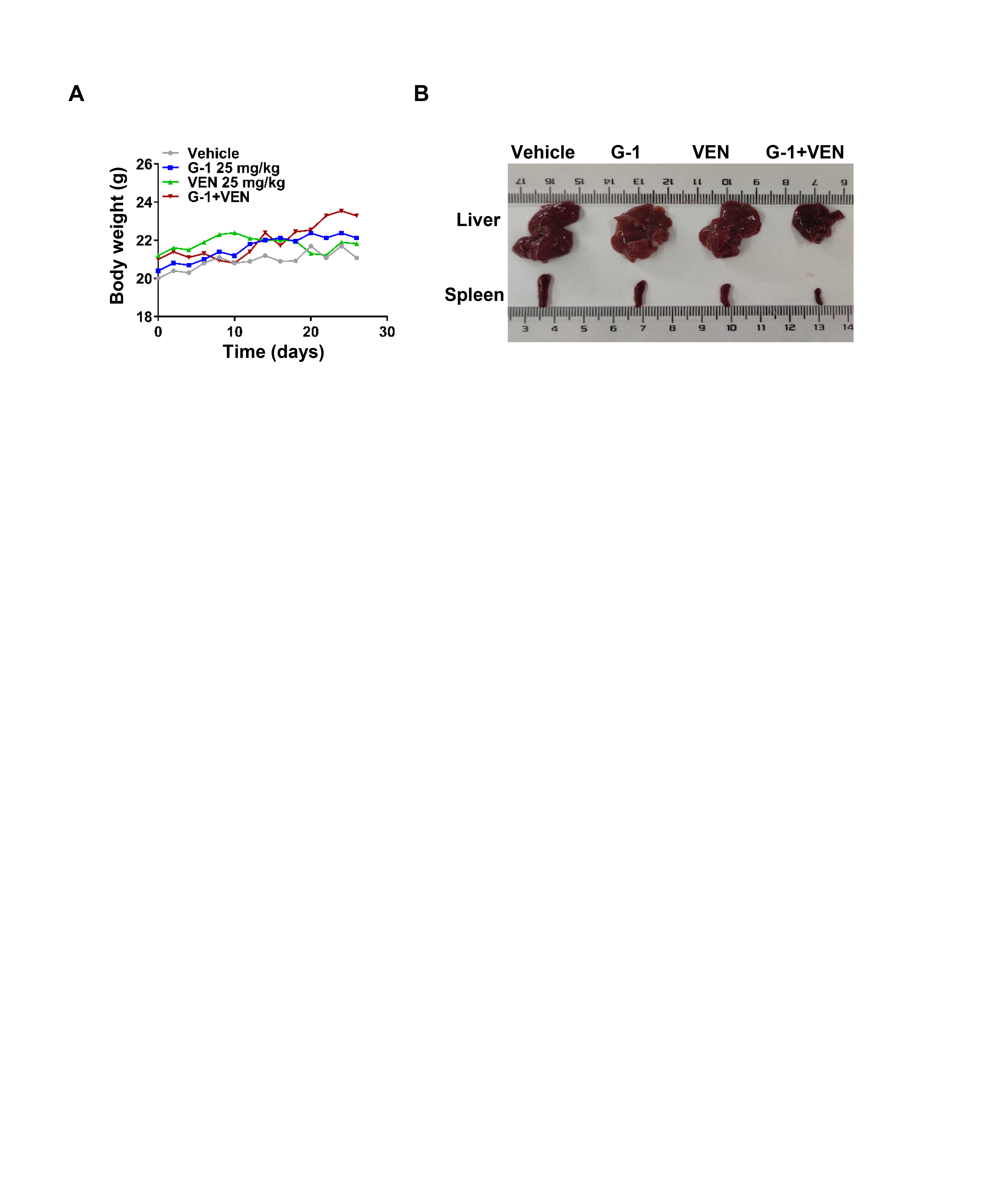


**Fig S8. Weights of mice and representative liver and spleen appearance.** (A) Average mouse body weights were measured every two days. (B) Images of livers and spleens appearance.
