# Supplementary material for: Targeted activation of GPER enhances the efficacy of venetoclax by boosting leukemic pyroptosis and CD8+ T cell immune function in acute myeloid leukemia
Source: Cell Death Dis. 2022 Oct 31;13(10):915. doi: 10.1038/s41419-022-05357-9 (PMC9622865; doi:10.1038/s41419-022-05357-9)
Supplement: Supplementary file 3 — Original Data File [file 41419_2022_5357_MOESM3_ESM.pdf]

**Figure. 1**

**D**

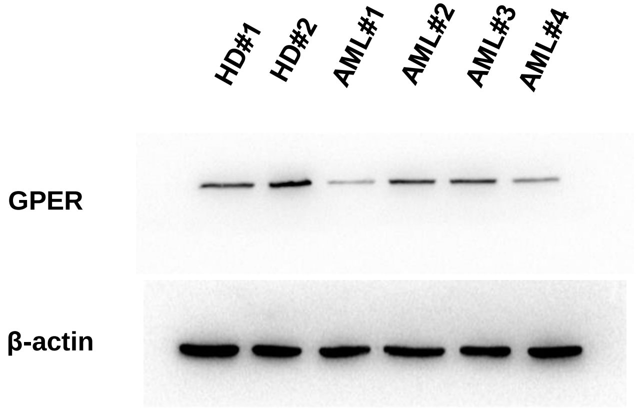
HD#1
HD#2
AML#1
AML#2
AML#3
AML#4
GPER
β-actin

**G**

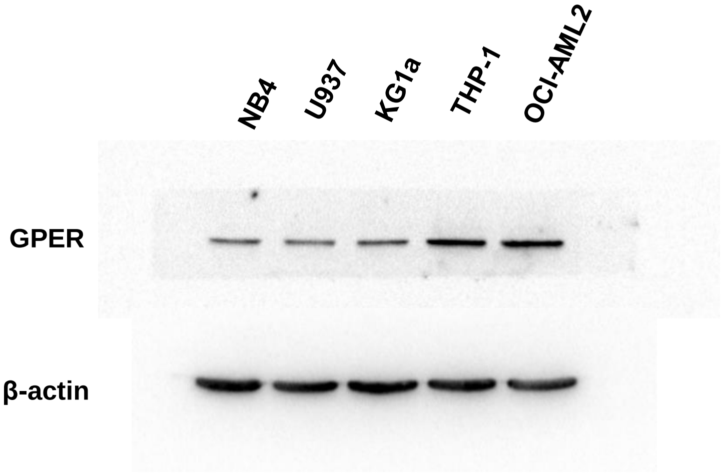
NB4
U937
KG1a
THP-1
OCI-AML2
GPER
β-actin

# Figure. 2

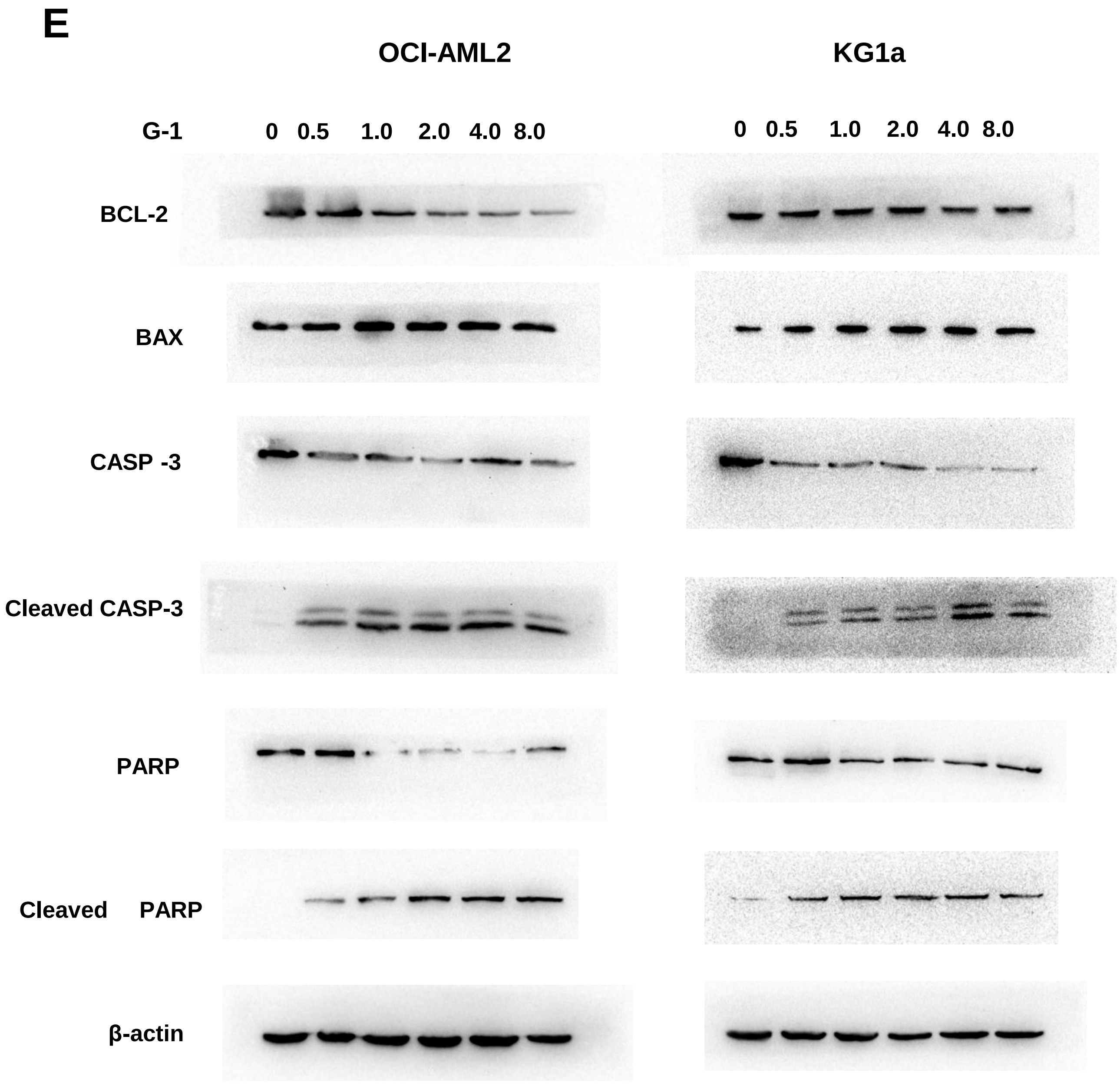
E
OCI-AML2
KG1a
G-1
0 0.5 1.0 2.0 4.0 8.0
0 0.5 1.0 2.0 4.0 8.0
BCL-2
BAX
CASP -3
Cleaved CASP-3
PARP
Cleaved PARP
β-actin

# Figure. 2

**F**

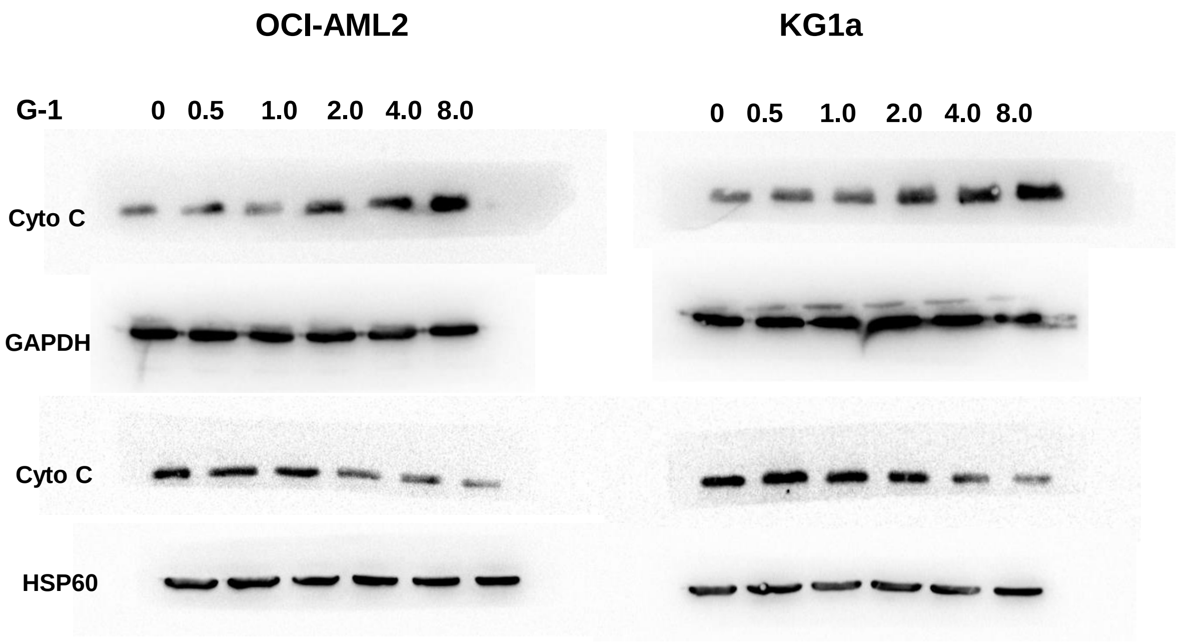
OCI-AML2
KG1a
G-1
0 0.5 1.0 2.0 4.0 8.0
0 0.5 1.0 2.0 4.0 8.0
Cyto C
GAPDH
Cyto C
HSP60

**G**

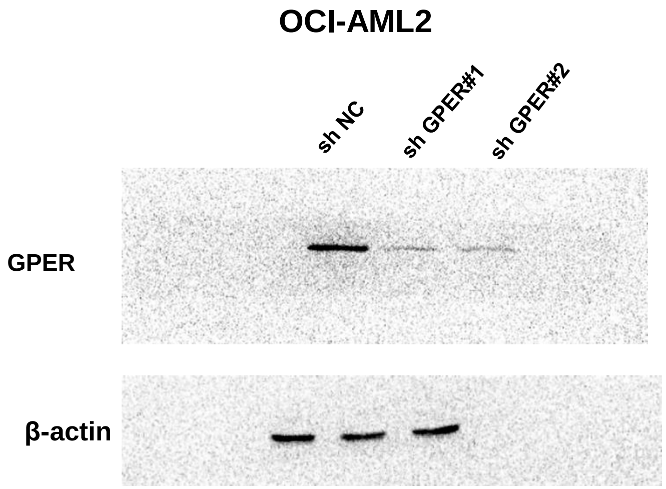
OCI-AML2
sh NC
sh GPER#1
sh GPER#2
GPER
β-actin

# Figure. 3

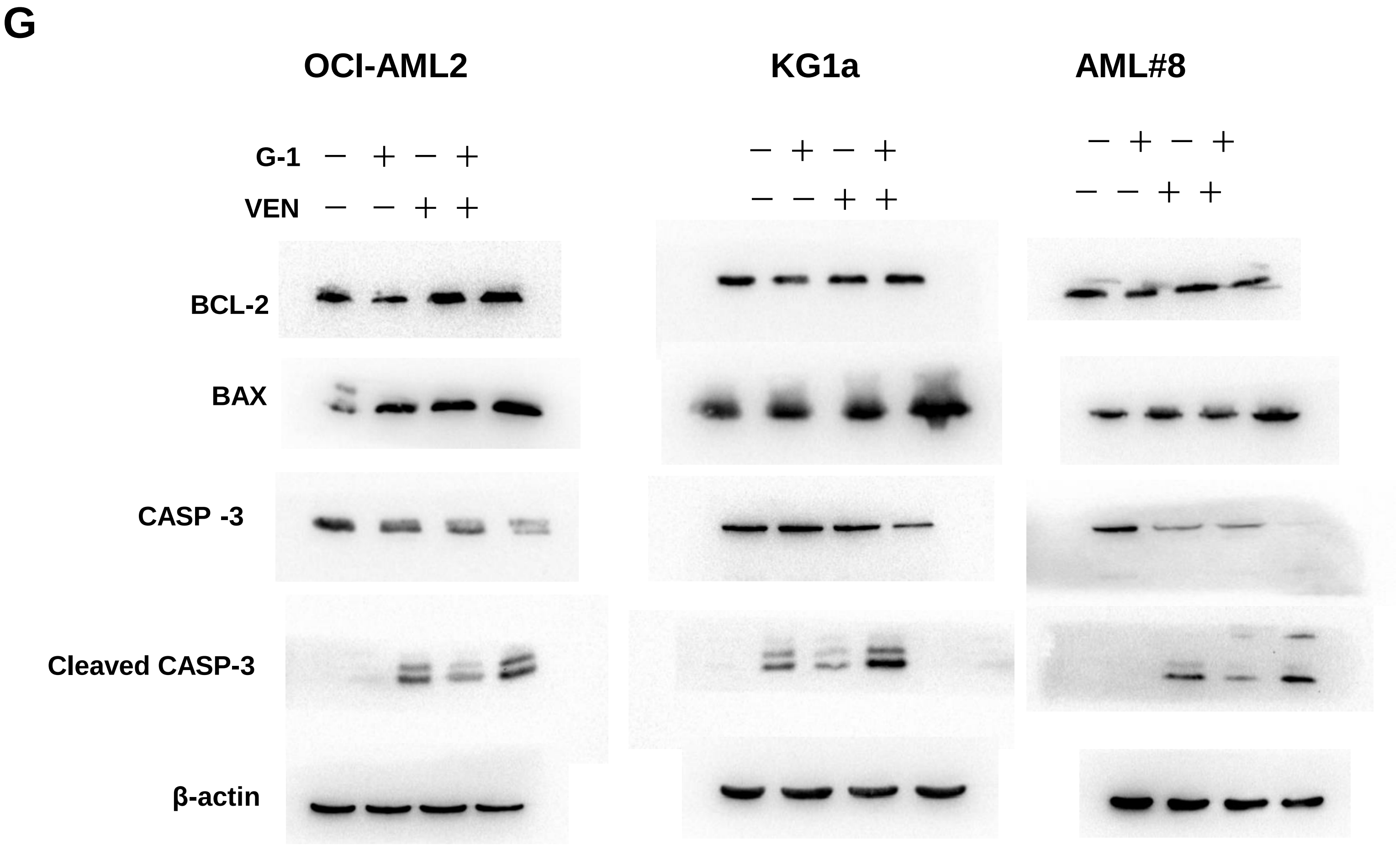
G
OCI-AML2
KG1a
AML#8
G-1 − + − +
VEN − − + +
− + − +
− − + +
− + − +
− − + +
BCL-2
BAX
CASP -3
Cleaved CASP-3
β-actin

# Figure. 3

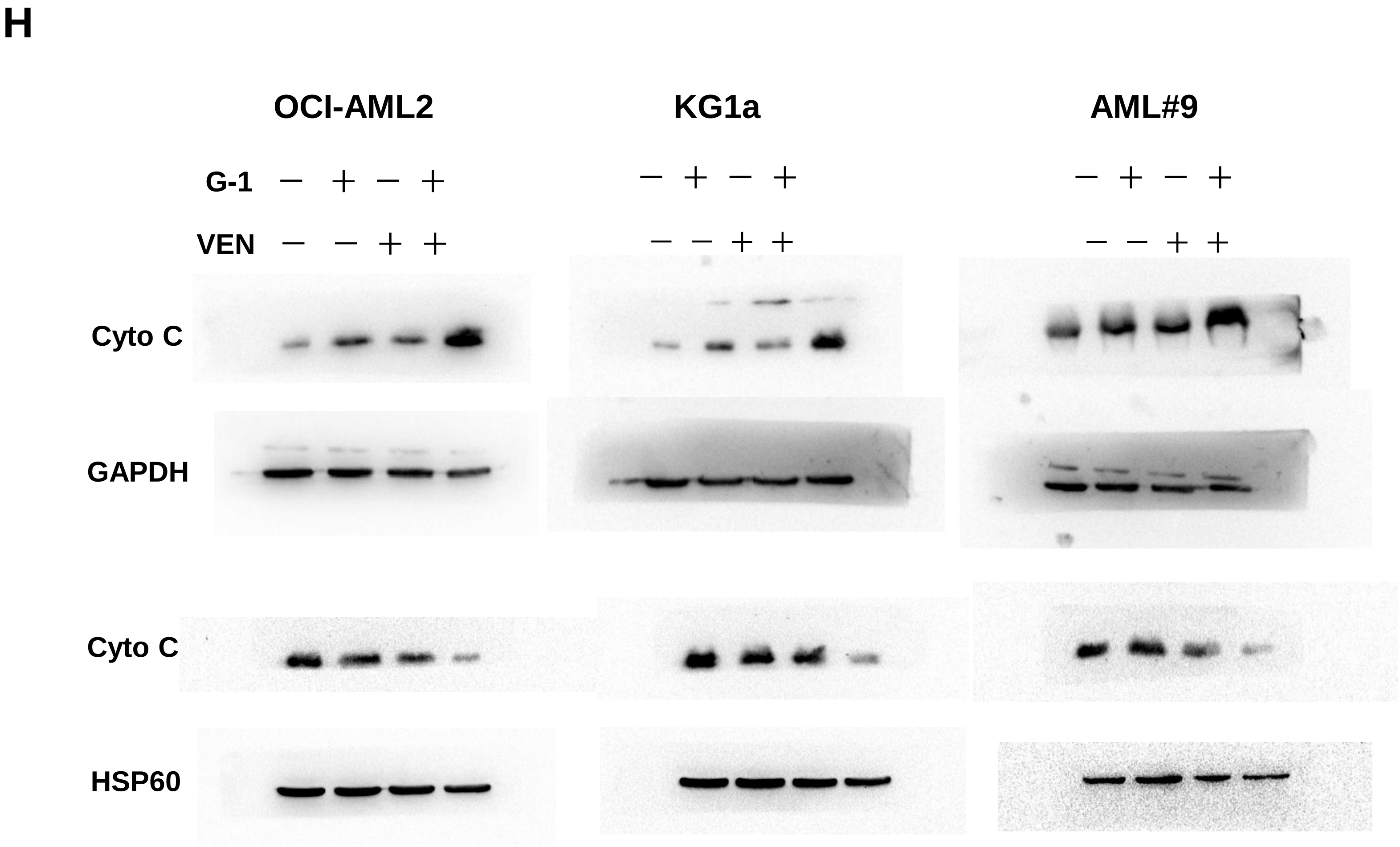

Figure. 5

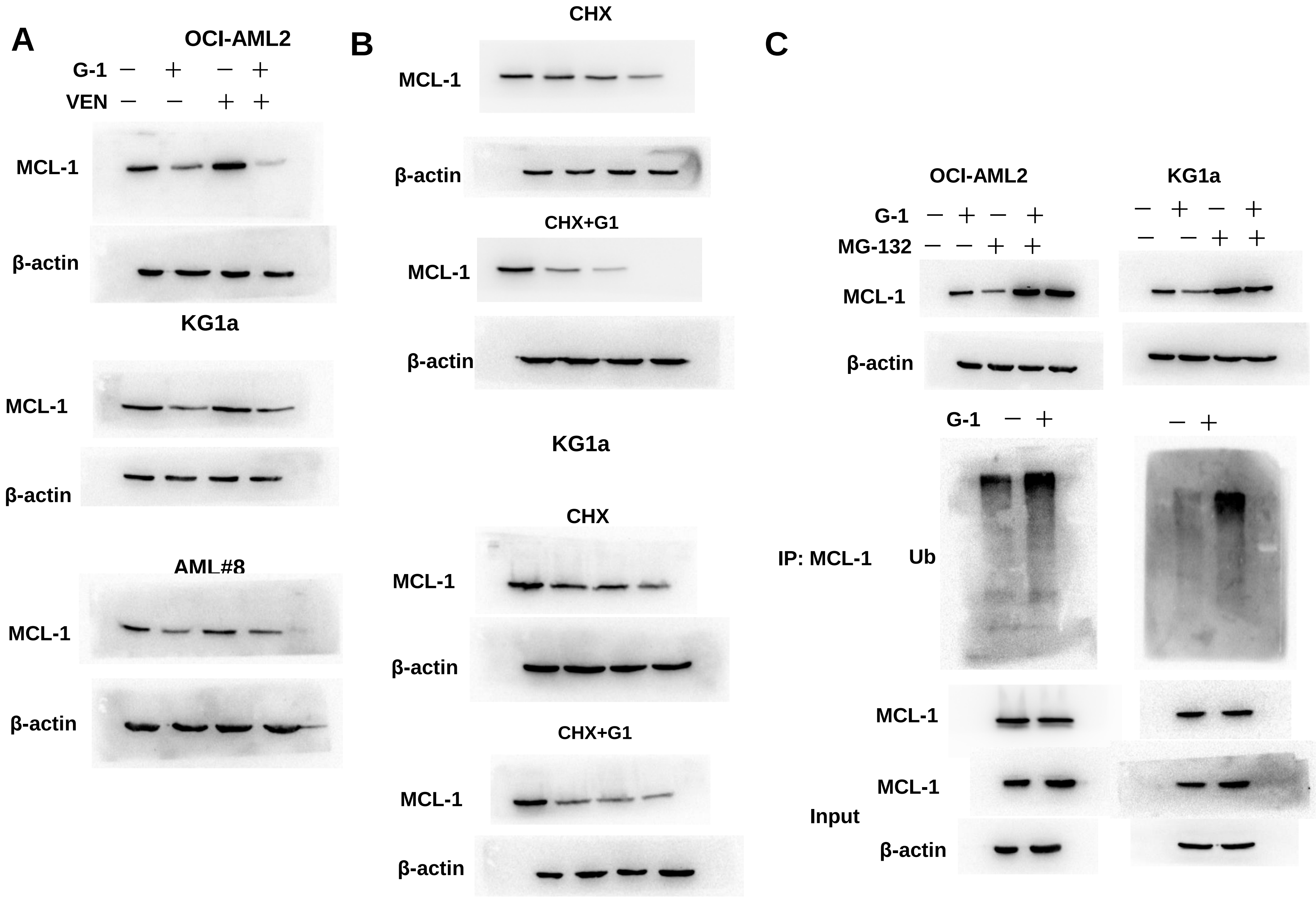
A
OCI-AML2
G-1 − + − +
VEN − − + +
MCL-1
β-actin
KG1a
MCL-1
β-actin
AML#8
MCL-1
β-actin
B
OCI-AML2
CHX
MCL-1
β-actin
CHX+G1
MCL-1
β-actin
KG1a
CHX
MCL-1
β-actin
CHX+G1
MCL-1
β-actin
C
OCI-AML2
KG1a
G-1 − + − +
MG-132 − − + +
MCL-1
β-actin
G-1 − +
IP: MCL-1
Ub
MCL-1
Input
MCL-1
β-actin

Figure. 5

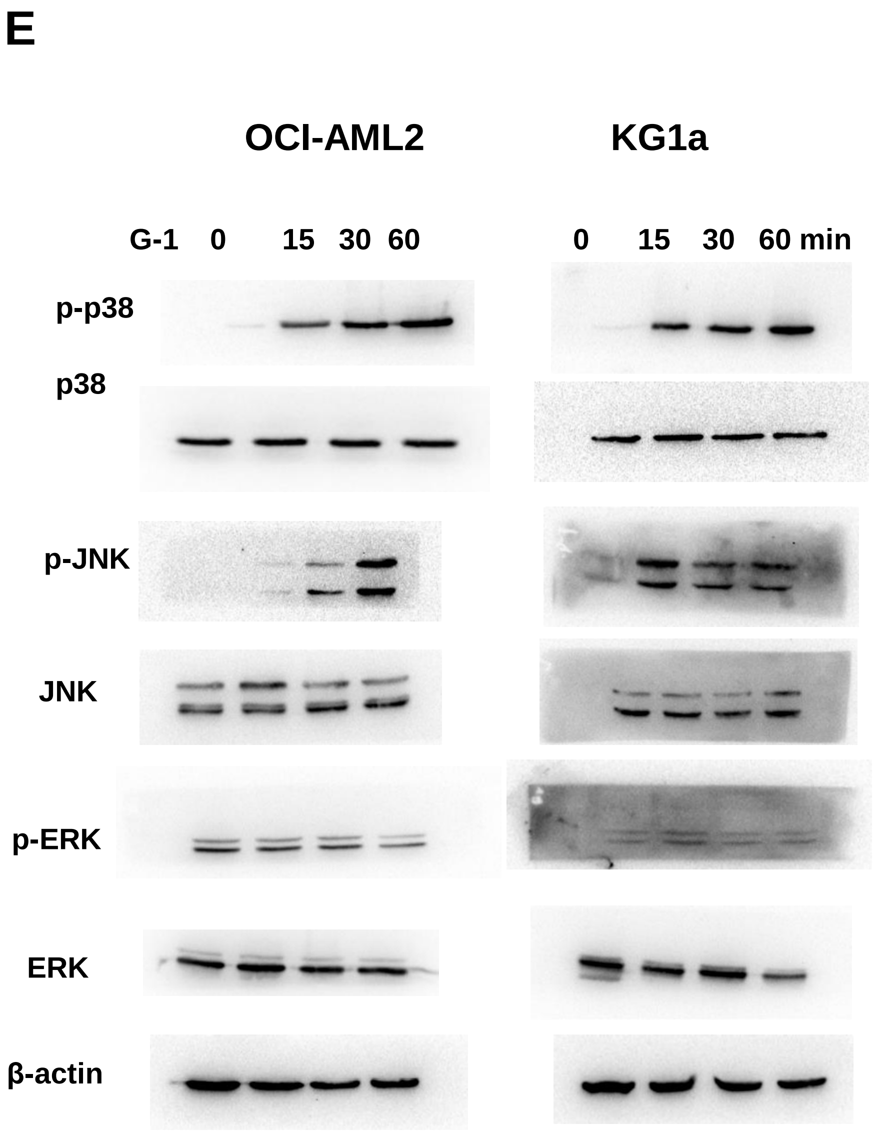
E
OCI-AML2
KG1a
G-1 0 15 30 60
0 15 30 60 min
p-p38
p38
p-JNK
JNK
p-ERK
ERK
β-actin

# Figure. 5

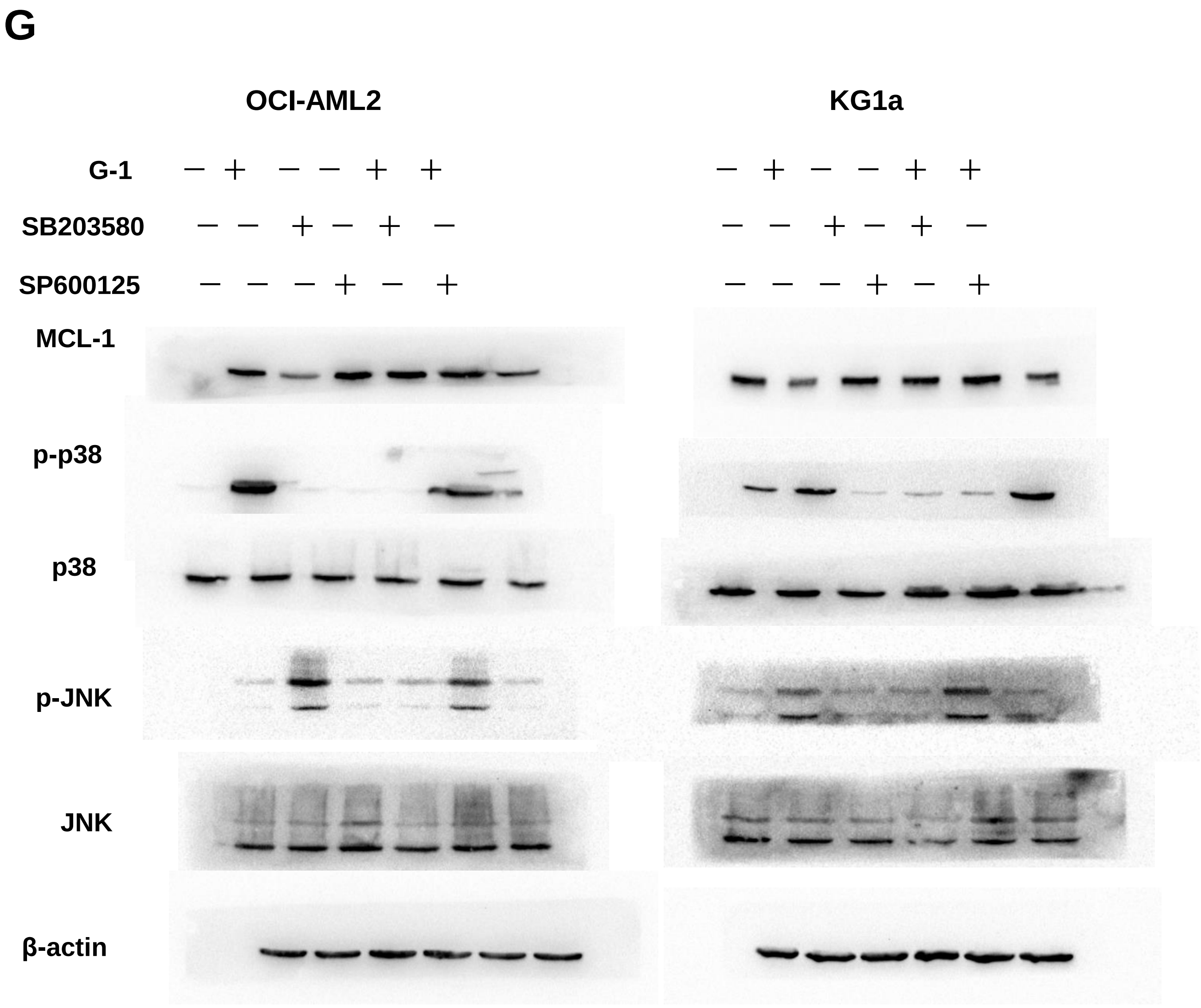
G
OCI-AML2
KG1a
G-1
SB203580
SP600125
MCL-1
p-p38
p38
p-JNK
JNK
β-actin

Figure. 5

H

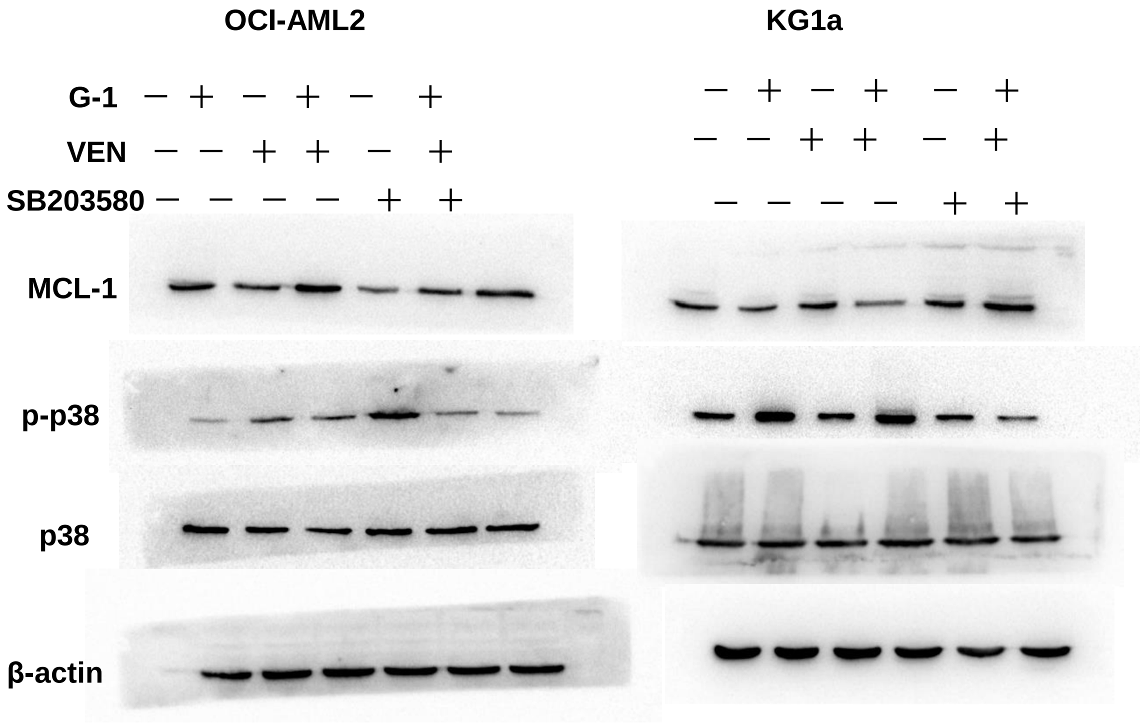

# Figure. 5

I

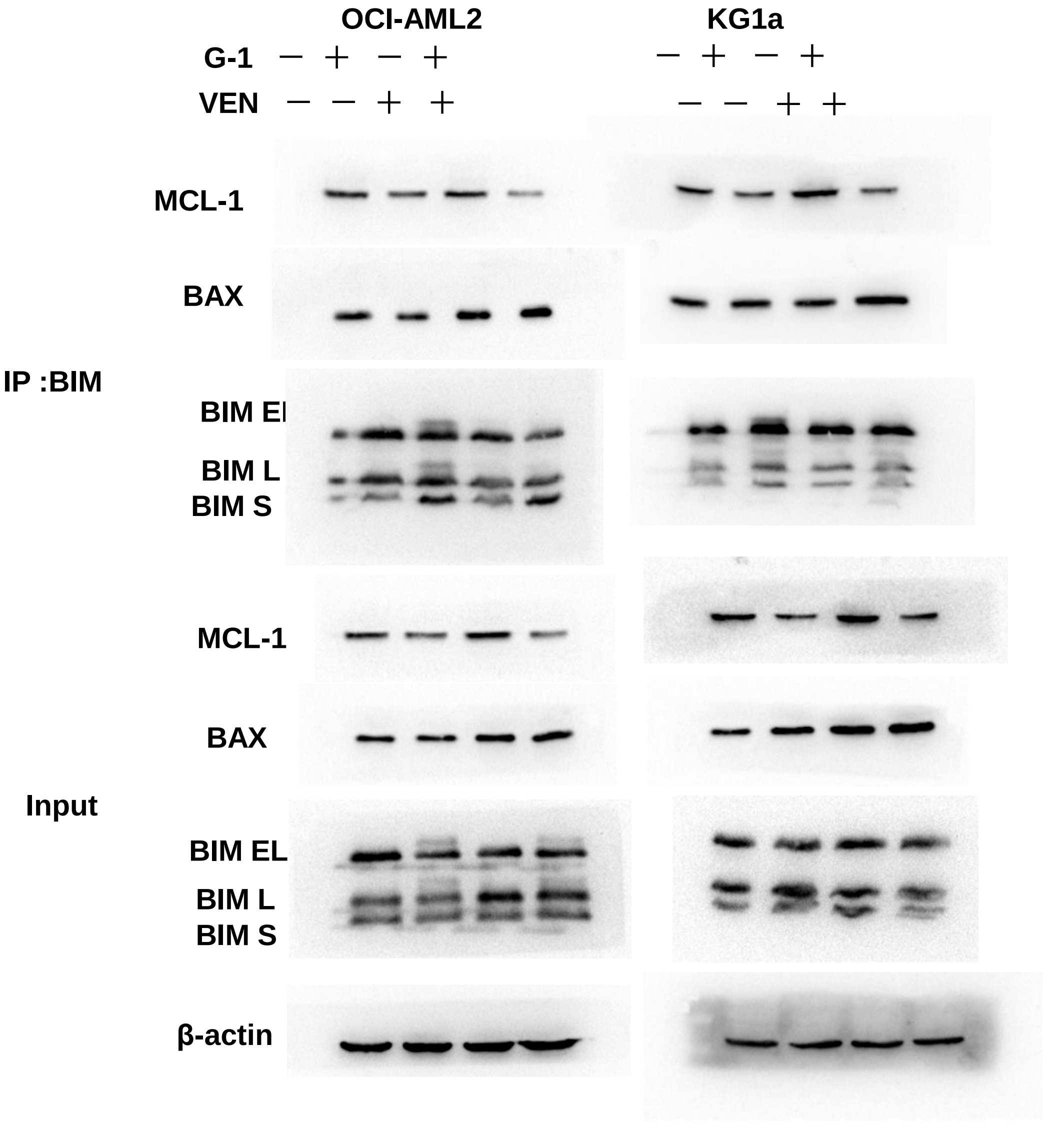

OCI-AML2
KG1a
G-1 − + − + − + − +
VEN − − + + − − + +
MCL-1
BAX
IP :BIM
BIM EL
BIM L
BIM S
MCL-1
BAX
Input
BIM EL
BIM L
BIM S
β-actin

Figure. 6

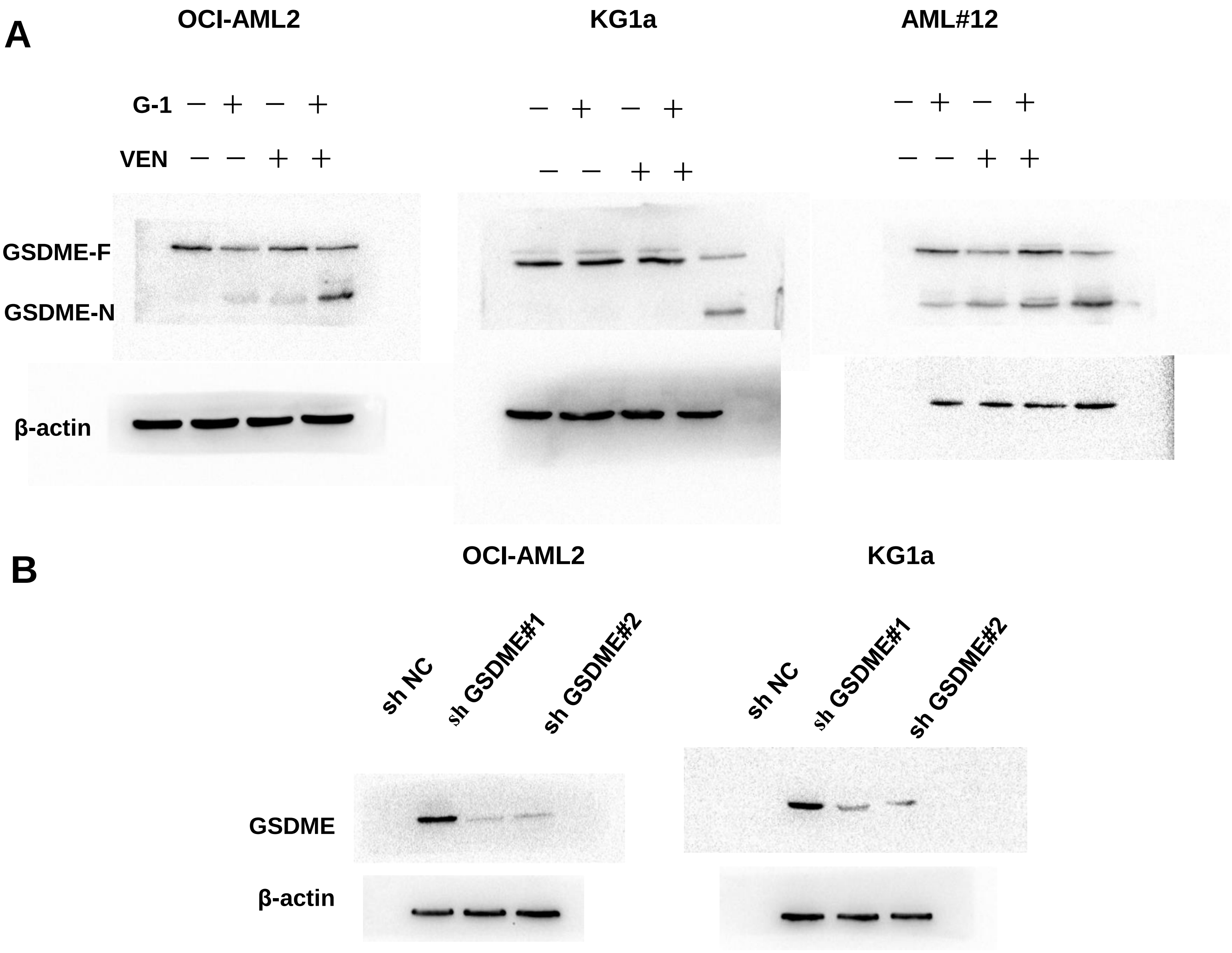
A
OCI-AML2
KG1a
AML#12
G-1 − + − +
VEN − − + +
− + − +
− − + +
− + − +
− − + +
GSDME-F
GSDME-N
β-actin
B
OCI-AML2
KG1a
sh NC
sh GSDME#1
sh GSDME#2
sh NC
sh GSDME#1
sh GSDME#2
GSDME
β-actin

Figure. 6

C

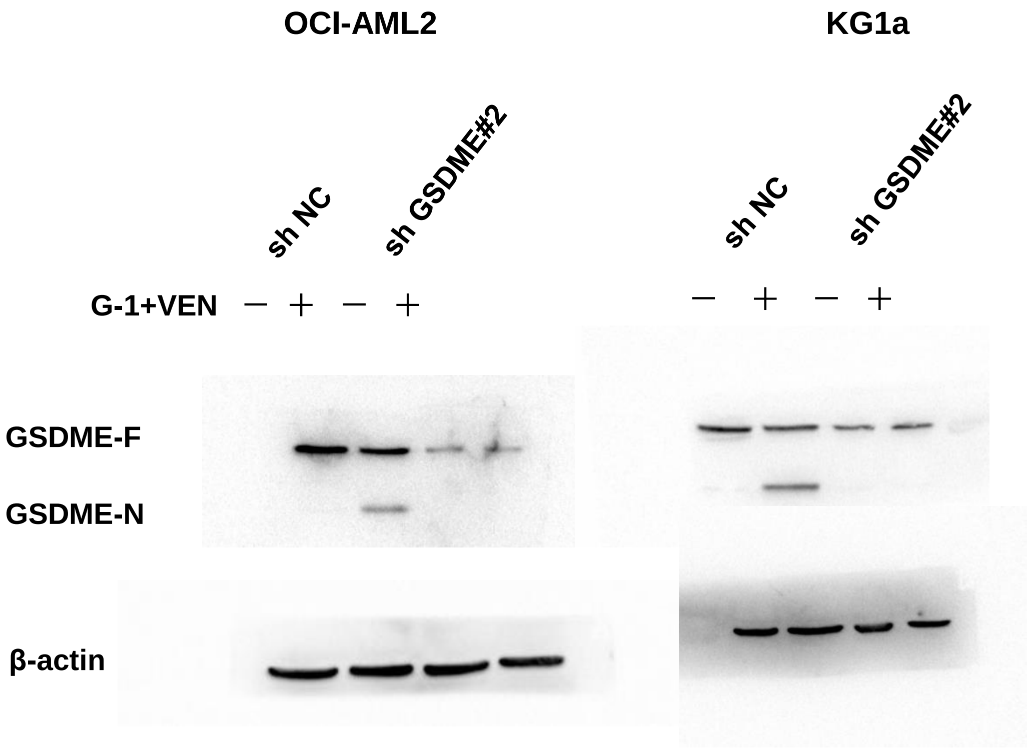
OCI-AML2
KG1a
sh NC
sh GSDME#2
sh NC
sh GSDME#2
G-1+VEN
−
+
−
+
−
+
−
+
GSDME-F
GSDME-N
β-actin

**Figure. 7**

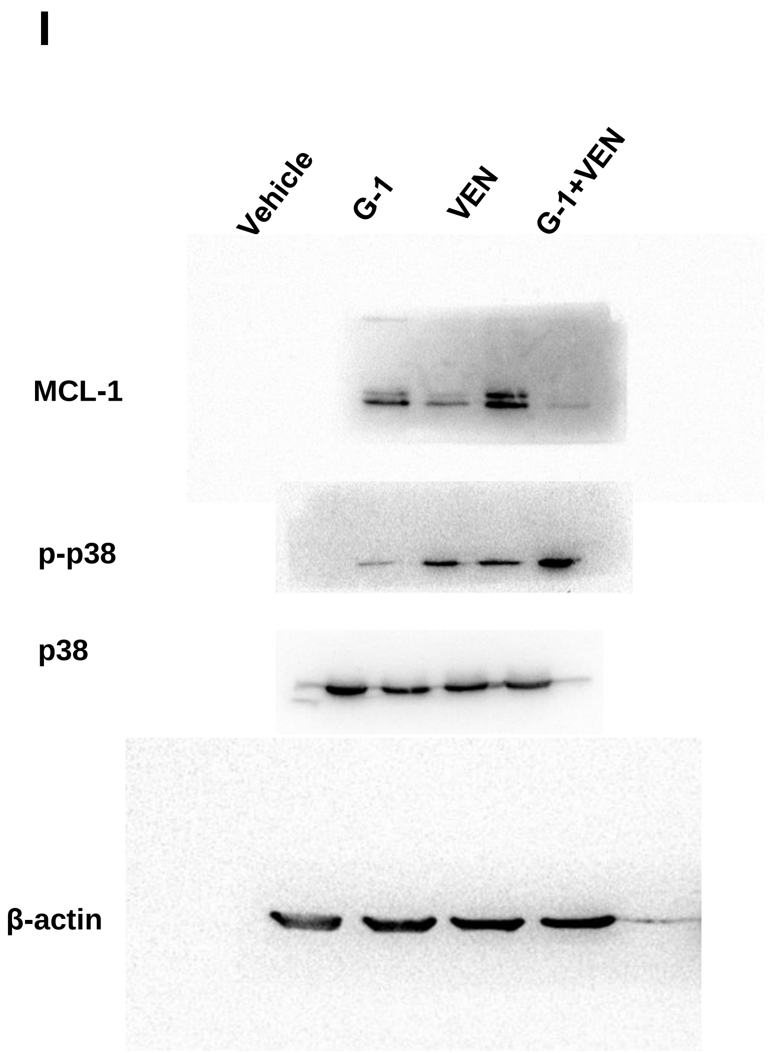
I
Vehicle
G-1
VEN
G-1+VEN
MCL-1
p-p38
p38
β-actin

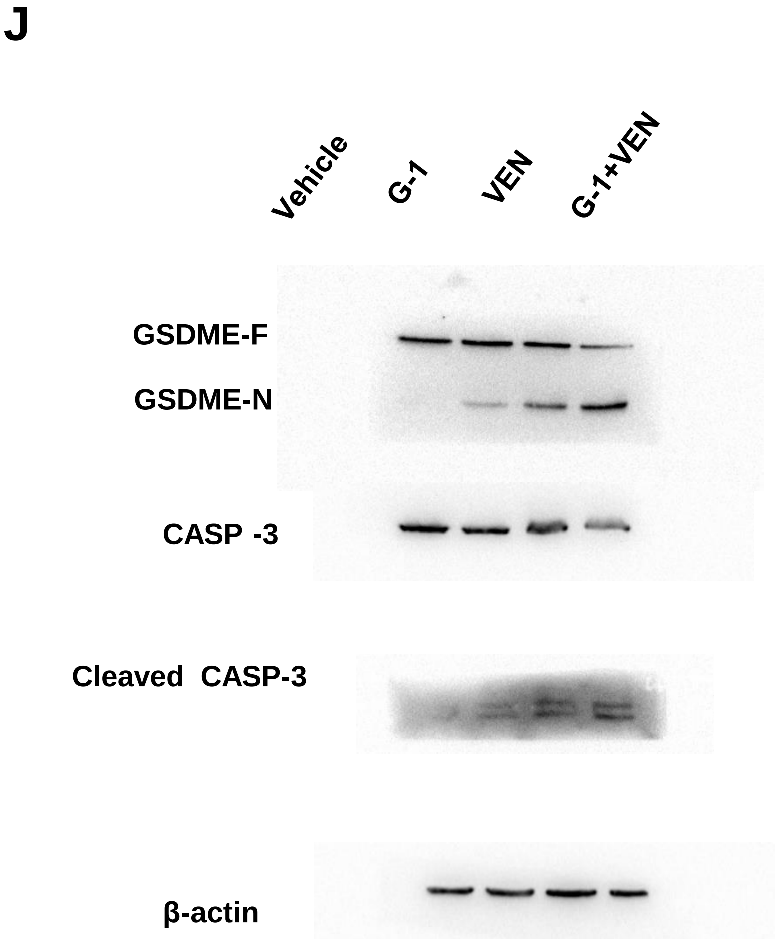
J
Vehicle
G-1
VEN
G-1+VEN
GSDME-F
GSDME-N
CASP -3
Cleaved CASP-3
β-actin

# Figure S1

## C

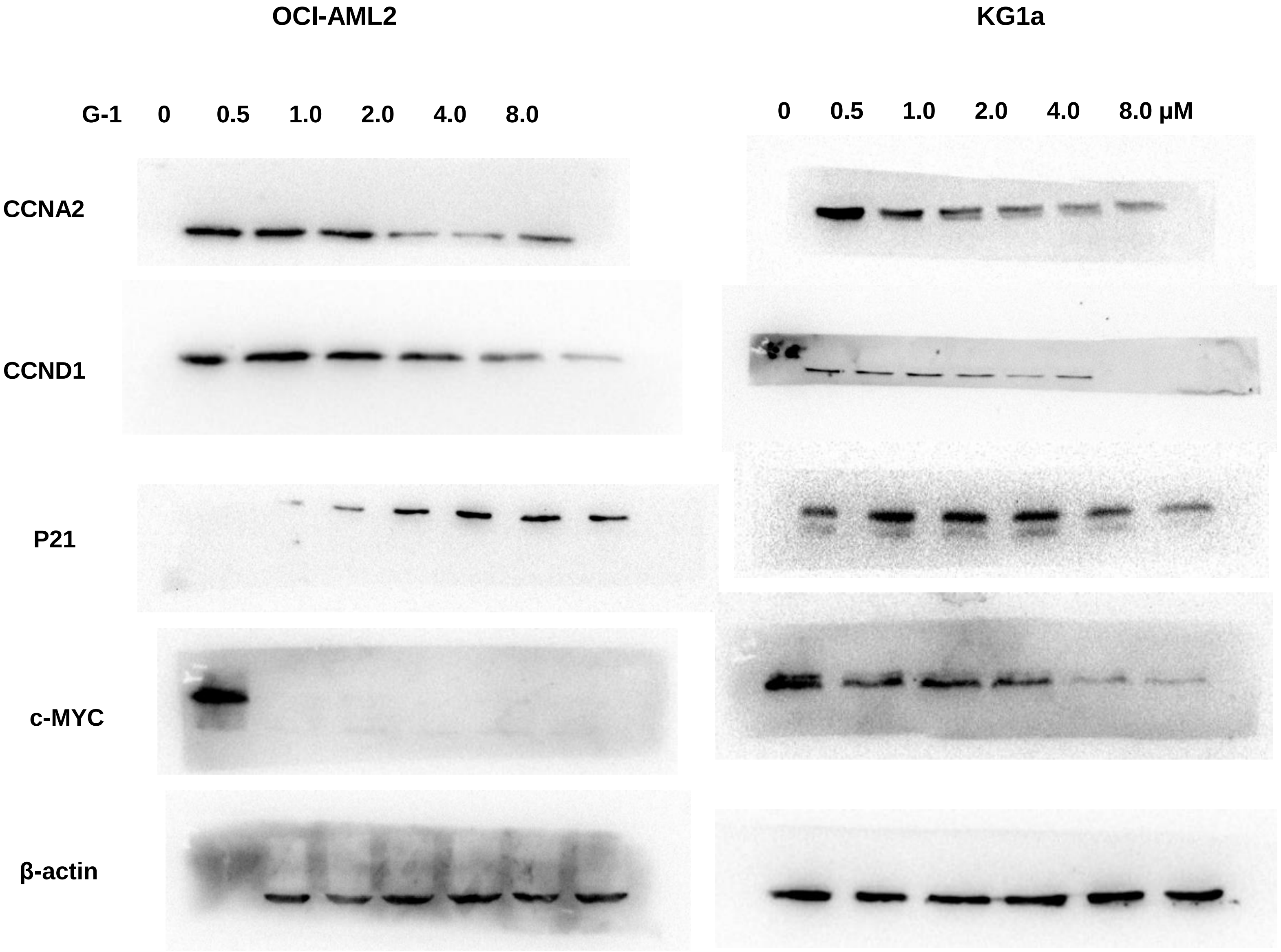

OCI-AML2
KG1a
G-1 0 0.5 1.0 2.0 4.0 8.0
0 0.5 1.0 2.0 4.0 8.0 μM
CCNA2
CCND1
P21
c-MYC
β-actin

# Figure S4

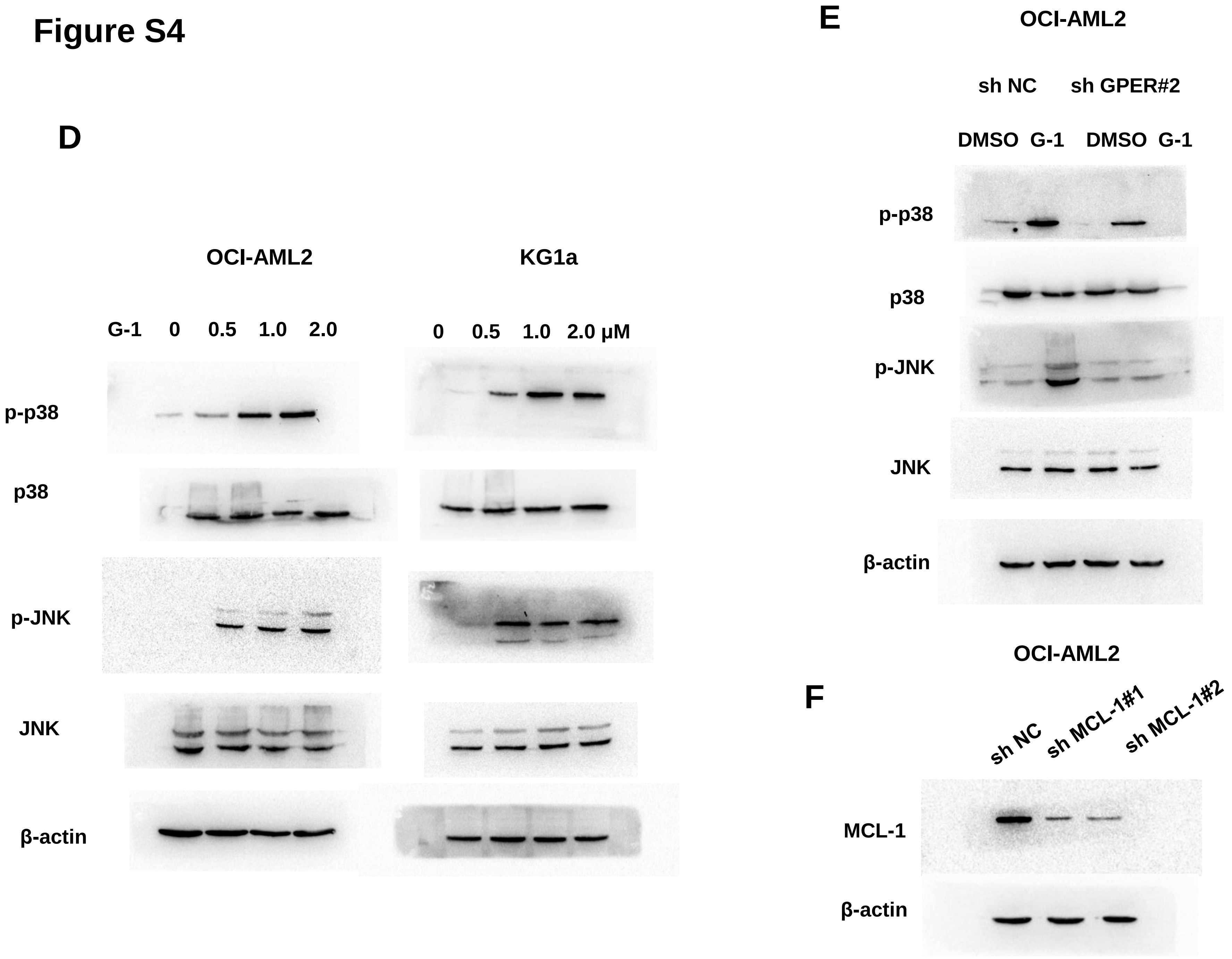
D
OCI-AML2
KG1a
G-1 0 0.5 1.0 2.0
0 0.5 1.0 2.0 μM
p-p38
p38
p-JNK
JNK
β-actin
E
OCI-AML2
sh NC sh GPER#2
DMSO G-1 DMSO G-1
p-p38
p38
p-JNK
JNK
β-actin
F
OCI-AML2
sh NC
sh MCL-1#1
sh MCL-1#2
MCL-1
β-actin

## Figure S5

B

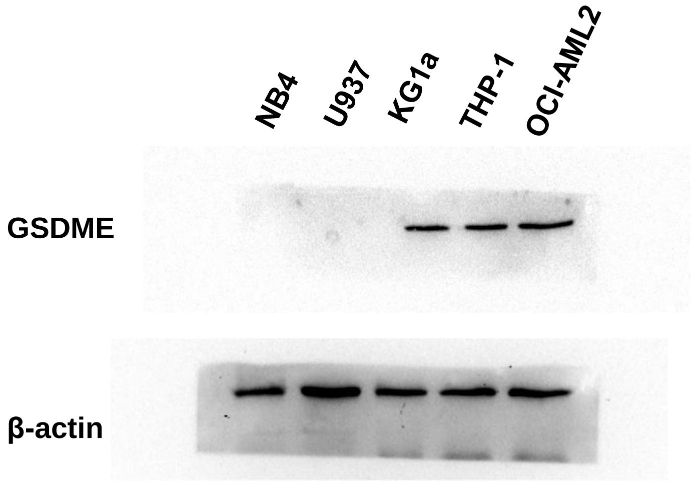
NB4
U937
KG1a
THP-1
OCI-AML2
GSDME
β-actin

C

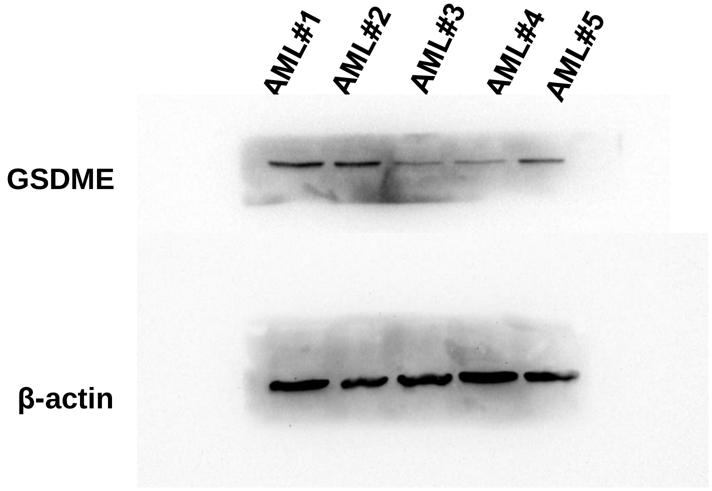
AML#1
AML#2
AML#3
AML#4
AML#5
GSDME
β-actin

# Figure S5

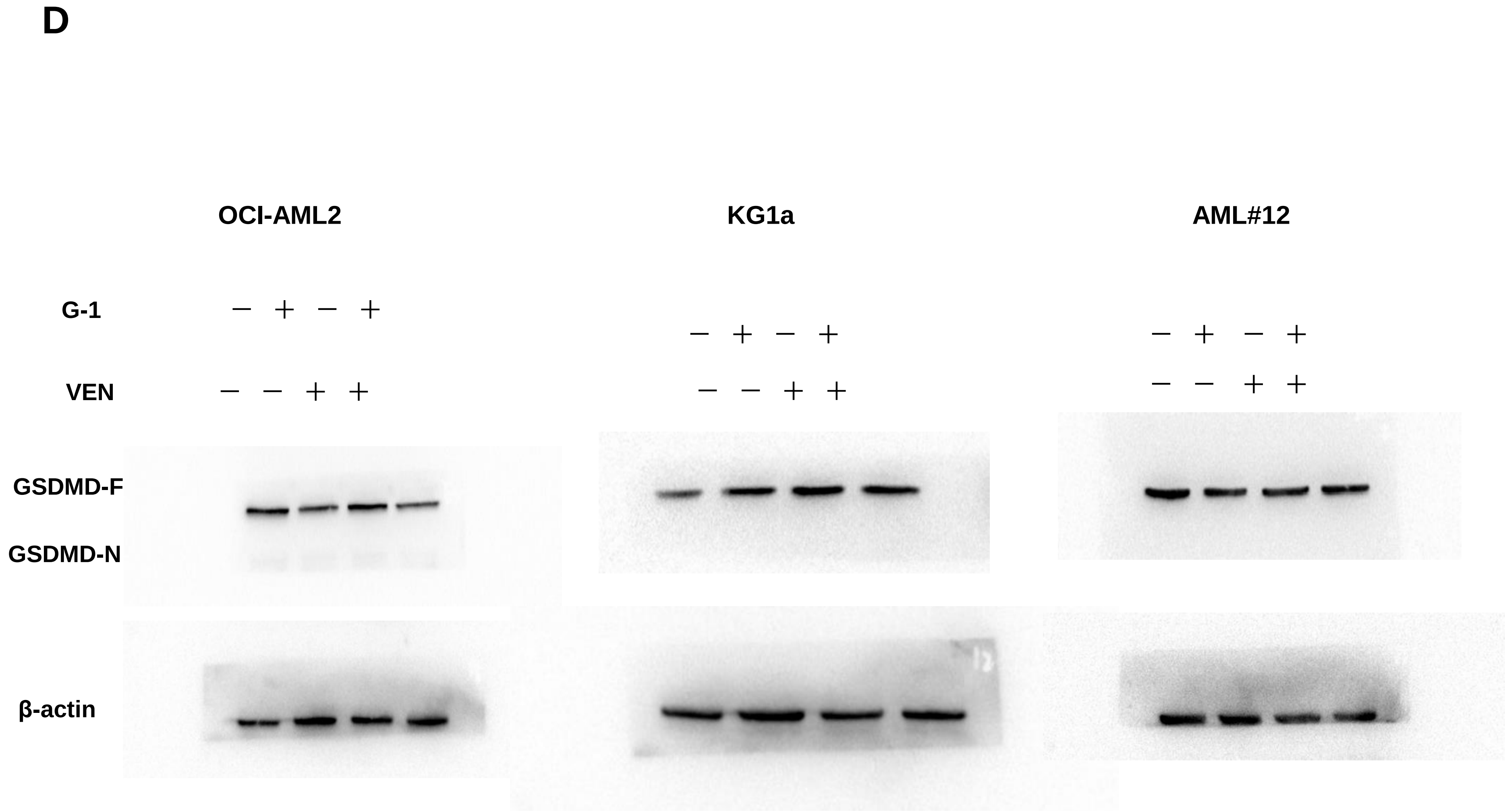

# Figure S6

**A**

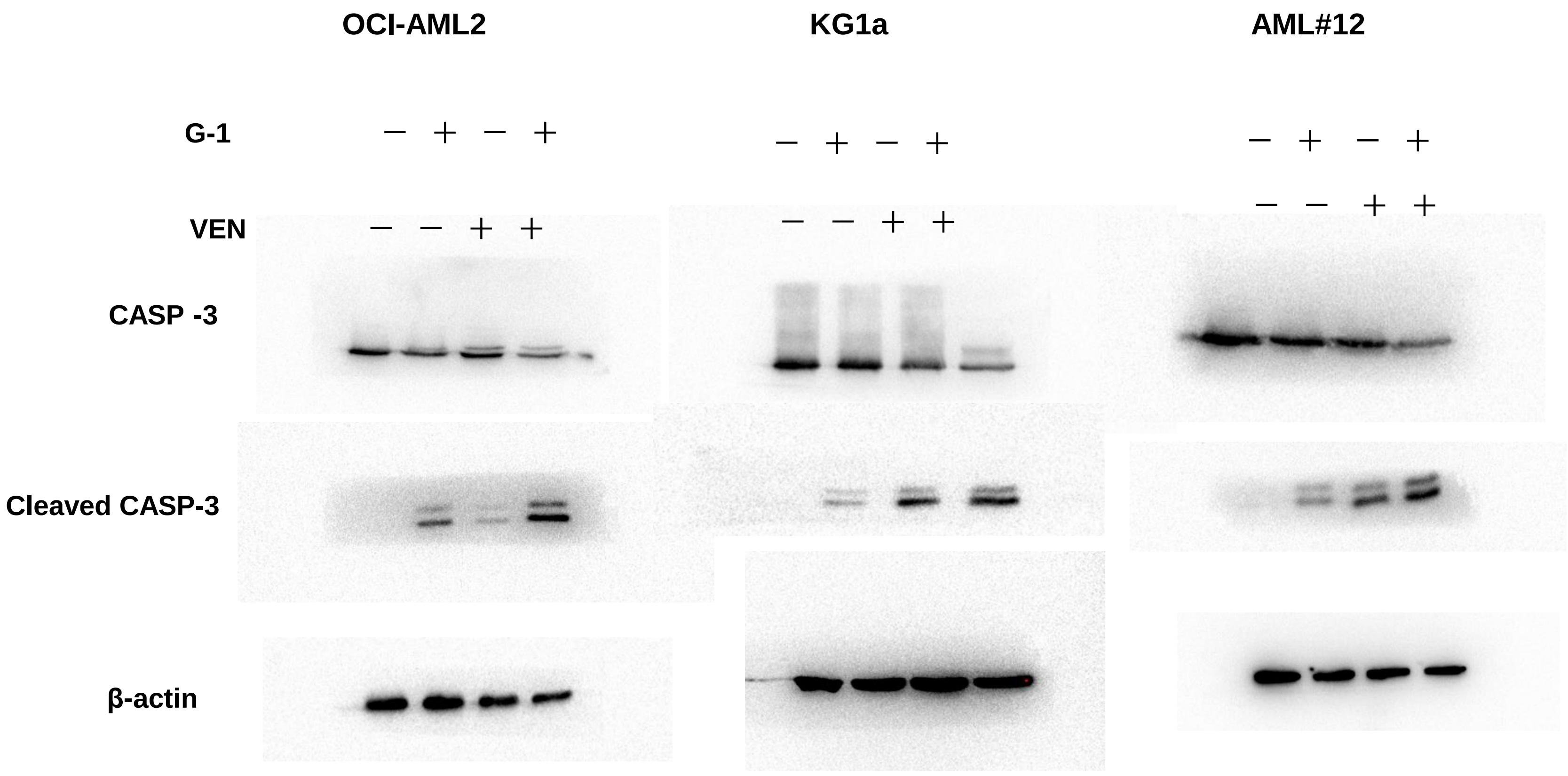

# Figure S6

## B

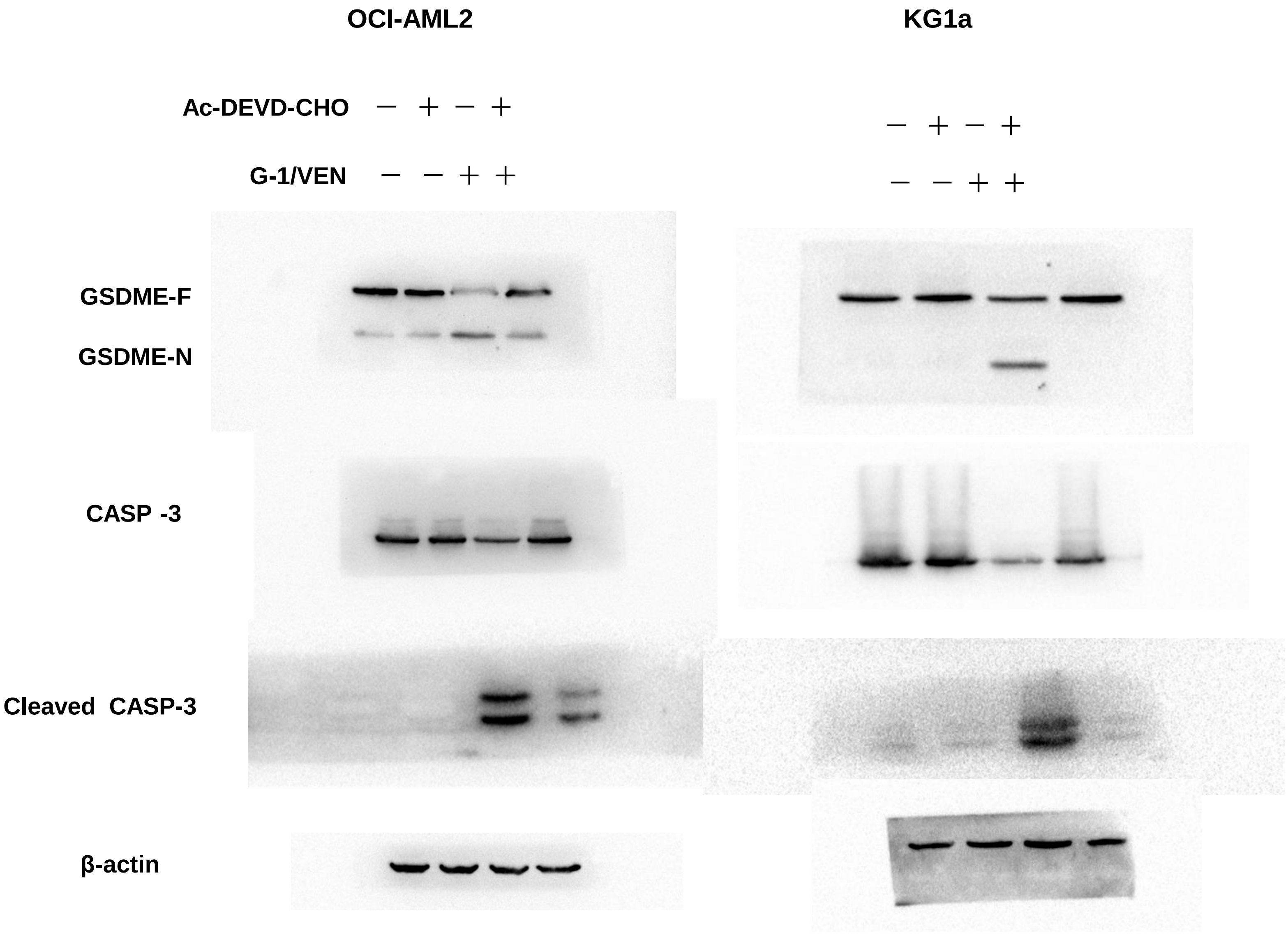

OCI-AML2
KG1a
Ac-DEVD-CHO − + − +
G-1/VEN − − + +
− + − +
− − + +
GSDME-F
GSDME-N
CASP -3
Cleaved CASP-3
β-actin

# Figure S7

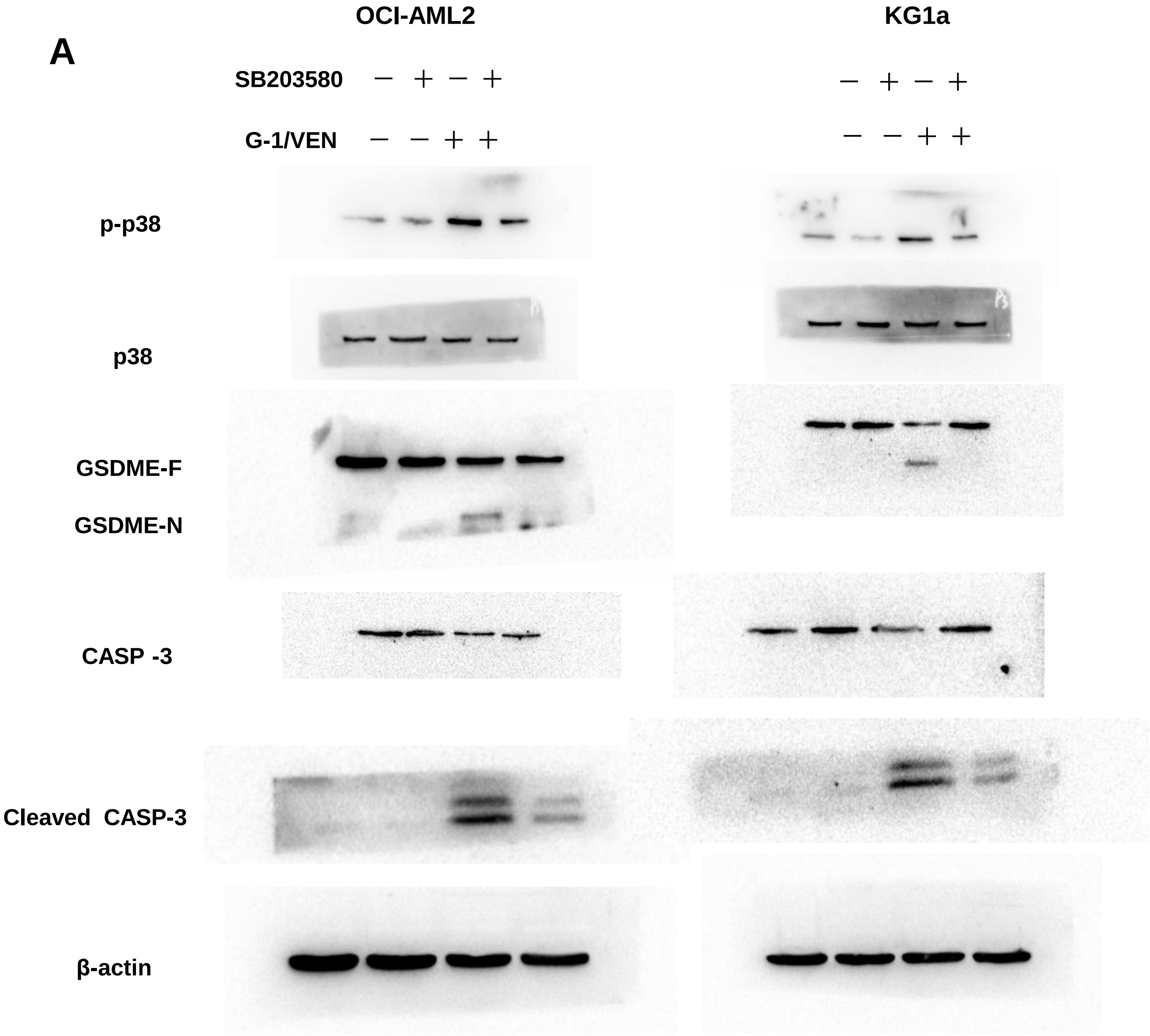
A
OCI-AML2
KG1a
SB203580
G-1/VEN
p-p38
p38
GSDME-F
GSDME-N
CASP -3
Cleaved CASP-3
β-actin
